# A Review of Enzyme-Induced Calcium Carbonate Precipitation Applicability in the Oil and Gas Industry

**DOI:** 10.3389/fbioe.2022.900881

**Published:** 2022-06-20

**Authors:** Sulaiman A. Alarifi, Ayyaz Mustafa, Kamal Omarov, Abdul Rehman Baig, Zeeshan Tariq, Mohamed Mahmoud

**Affiliations:** ^1^ Department of Petroleum Engineering, College of Petroleum Engineering and Geosciences, King Fahd University of Petroleum and Minerals (KFUPM), Dhahran, Saudi Arabia; ^2^ Center for Integrative Petroleum Research (CIPR), College of Petroleum Engineering and Geosciences, King Fahd University of Petroleum and Minerals, Dhahran, Saudi Arabia; ^3^ Physical Science and Engineering Division, King Abdullah University of Science and Technology (KAUST), Thuwal, Saudi Arabia

**Keywords:** EICP, sand consolidation, calcium carbonate precipitation, enzymes, bio-cemented sand

## Abstract

Enzyme-induced calcium carbonate precipitation (EICP) techniques are used in several disciplines and for a wide range of applications. In the oil and gas industry, EICP is a relatively new technique and is actively used for enhanced oil recovery applications, removal of undesired chemicals and generating desired chemicals *in situ*, and plugging of fractures, lost circulation, and sand consolidation. Many oil- and gas-bearing formations encounter the problem of the flow of sand grains into the wellbore along with the reservoir fluids. This study offers a detailed review of sand consolidation using EICP to solve and prevent sand production issues in oil and gas wells. Interest in bio-cementation techniques has gained a sharp increase recently due to their sustainable and environmentally friendly nature. An overview of the factors affecting the EICP technique is discussed with an emphasis on the *in situ* reactions, leading to sand consolidation. Furthermore, this study provides a guideline to assess sand consolidation performance and the applicability of EICP to mitigate sand production issues in oil and gas wells.

## 1 Introduction

The use of biotechnology has led to a great advancement in scientific and industrial progress in recent years. Many economically significant industries such as food processing, agriculture, and pharmaceutical have implemented solutions driven by biotechnology (Springham et al., 1991). Ultimately, such technology contributed greatly to improving the quality of life for humankind. Biotechnology is a broad area of biology that involves the use of living systems and organisms to develop or make products. The term biotechnology was introduced in 1919 by Karl Ereky and was defined as the production of products from raw materials with the aid of living organisms (Ereky, 1919).

Biological systems, including microorganisms and enzymes, have been used widely in recent years in the oil and gas industry for ultimately improving hydrocarbon recovery. Most of the applications of biotechnology in the oil and gas industry involve the use of enzymes in either degrading unwanted chemicals or producing desired chemicals (Harris and McKay, 1998). Degrading unwanted chemicals involves, for instance, the degrading and reduction of viscosity of cellulose-containing fluid during well completion, workover, and fracturing operations (Tjon Joe Pin and Beall, 1996). The production of desired chemicals includes the production of useful chemicals *in situ*, such as calcium carbonate, which can be produced by an enzyme-based process that could lead to sand consolidation. Recently, many applications of enzymes are becoming widely popular in the oil and gas industry, such as microbial enhanced oil recovery, which revolves around the use of bacteria to enhance recovery and was first introduced in 1946 by Zobell (1946).

**FIGURE 1:**
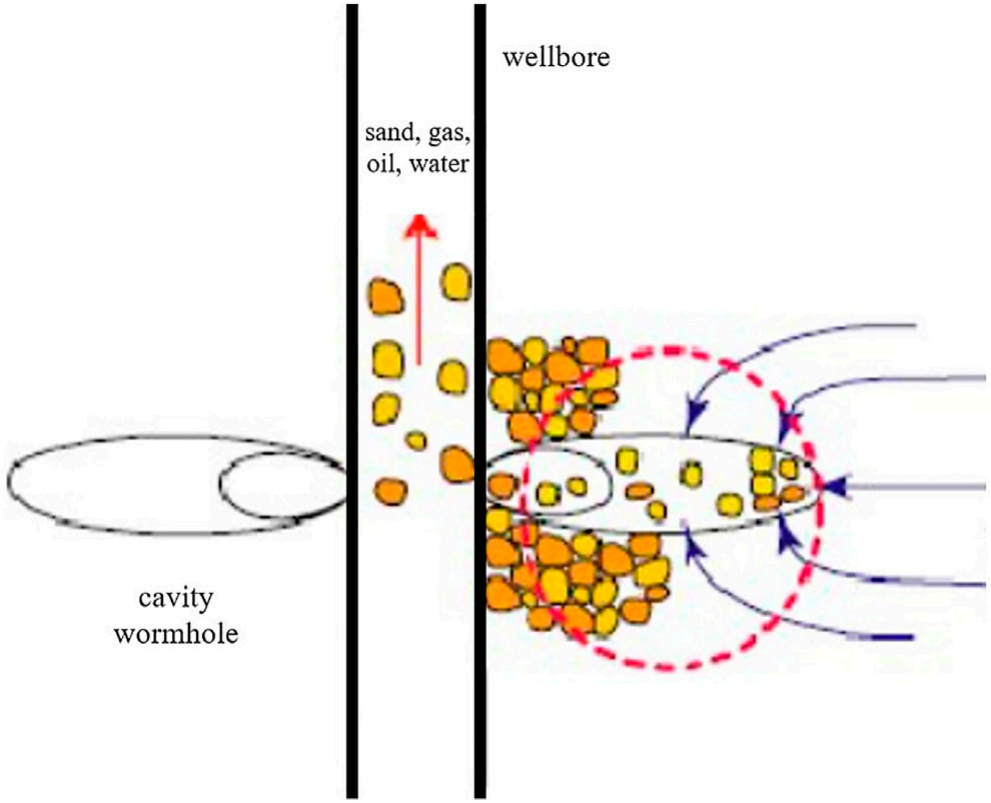
Sand production process.

Many oil- and gas-bearing formations encounter the problem of the flow of sand grains into the wellbore with reservoir fluids (Patton and Ablott, 1981) (Figure 1). The problems are more severe in unconsolidated sandstone reservoirs. Numerous problems may occur due to the influx of sand into the wellbore, such as liner plugging, accumulation of sand in separators, and erosion of pipelines and valves. The oil industry loses a substantial amount of revenue due to the decrease in the production rate and spends a huge amount of money on repairing and cleaning tools every year. Moreover, the major safety risk is also associated with the erosion of tools and valves, especially in high-pressure reservoirs. Hence, the sand production problem has been under research for many years (Suman et al., 1983).

The sand production problem is caused due to shear or tensile failure in low compressive strength formations because of high shear stresses and high drag forces. Drop-in wellbore pressure and frictional forces cause the flow of sand grains into the wellbore (El-Sayed et al., 2001). These solids gather in the wellbore and hinder the flow of formation fluid, resulting in significant reduction in production (Hower and Ramos, 1957; King and Harry, 1964; Butler, 1966). The sand particles are also responsible for damaging the equipment and tools such as pumps, pipes, wellheads, valves, and chokes (Mishra and Ojha, 2015). Sand production from oil wells is an ongoing challenge for the oil industry.

When the sand production is so severe, then intervention is required for remedial sand control or new sidetracks are required. Several methods have been suggested and implemented to control sand production from oil-producing formations (Doson, 1922; Scott, 1928; Buckley and Wrightsman, 1945). The intrusion of sand grains into the wellbore during oil production may be prevented or reduced by using the controlling methods. Generally, mechanical, chemical processes, or a combination of both are used to control sand production. Mechanical practices include sand screens, slotted or perforated lines, gravel packing, and filters (Wrightsman, 1949; Cardwell and Ritch, 1950), whereas chemical processes require the injection of chemical reagents such as epoxy, plastics, resins, and phenols into the unconsolidated subterranean formations (Brooks, 1971; Burger et al., 1979; Burger et al., 1986). Chemical processes assist in binding the sand grains and enhance the formation strength without hindering the flow paths within the formation. Sometimes, selective completion and production practices are followed to control the sand movement into the wellbore. Each of these methods is successful for limited applications (El-Sayed et al., 2001).

The objective of this study is to investigate the applicability of utilizing the environmentally friendly enzymatic-assisted calcium carbonate (CaCO_3_) precipitation for sand control applications by *in situ* precipitation of calcium carbonate between loose sand grains in high-temperature reservoirs. The precipitation mechanism, reaction, and several performance assessment techniques are investigated and discussed. In addition, the effect of different additives, pH, and temperature is investigated. The performance assessment methods discussed include using uniaxial compressive strength, measuring permeability, triaxial compression tests, and molecular dynamic simulation.

## 2 Enzyme Applications in the Oil and Gas Industry

### 2.1 Enhanced Oil Recovery

Studies have shown that enhanced oil recovery (EOR) processes can be applied at any production stage and that different EOR methods exist. Conventionally, EOR is usually implemented after secondary recovery (Luque Alanis et al., 2015). In recent years, researchers have examined enzyme potential in EOR due to their eco-friendly nature, renewable sources, biodegradable nature, and workability in salty and hot environments. In this section, we review some earlier studies on enzyme applications in EOR.

Feng et al. (2007) described their use of enzymes in laboratory experiments and reservoir field tests. They performed core flooding experiments on 7-day aged core samples using 3, 6, and 10% improved enzyme concentrations. Their model displacement experiments showed improved recovery rates of 12.4–16.3%, 13.9–20%, and 15.7–21.1%, respectively. They also looked at the potential of upgraded enzymes to enhance EOR. The use of a modified enzyme resulted in improved oil mobility and the emulsification process was favored by the conversion of oil-wet sections into water-wet segments.

In addition, Nasiri et al. (2009) studied the EOR potential of enzymes by conducting spontaneous imbibition and core flooding experiments with seawater and Berea sandstone. They conducted two flooding tests in the tertiary mode with 1% enzyme-brine injection on two different core samples. The first core proved to have an oil recovery of 42% OOIP with 10-pore volume (PV) water injection and an increase of 11% with 34 PV injection. From the second core, recovery of 47% (Original Oil in place) was recorded with a water injection of 10 PV, and recovery escalated to 3.5% with a 40 pore volume injection. Based on the observed core behavioral difference, this difference was due to the variance in wet conditions. Oil production was mainly observed prior to water breakthrough at 0.37 pore volume for the first core while after the breakthrough at 0.33 pore volume for the second core. Additional oil production was noticed, despite the fact that most of the oil was generated prior to the breakthrough. Because of the spontaneous imbibition experiments, enzyme-brine system imbibition was delayed compared with that of raw brine, but the former exhibited more total oil production rate than the untreated brine of 2% OOIP.

Wang et al. (2008) conducted three core flooding experiments on three different handmade cemented rock samples using three enzyme concentrations (1, 2, and 5%). The oil was recovered from flooding in 90–95% of cases, and there was no significant recovery difference among the three enzyme concentrations; however, the most recovery was accomplished with 5%.

There have also been studies on the potential of enzyme EOR for low-permeability tight formations characterized by oil recovery below 10% (Salahshoor et al., 2018). Using spontaneous imbibition tests, Salahshoor et al. (2019) examined two Berea sandstone outcrops and eight Woodford shale outcrops for enzyme EOR (EEOR). An enzyme solution with 10 wt.% concentration was compared with deionized water imbibition to determine oil recovery results in sandstone and shale. The results indicated that the solution of the enzyme with 10 wt.% concentration retrieved 50% and 10–20% oil relative to deionized water. They noticed no notable differences in the enzyme EOR performance in either carbonate or clay-rich rocks studied, but they found that 5 wt.% enzyme concentration performs better in shale than 10 wt.%.

An emerging EOR technique, enzyme-enhanced oil recovery (EEOR), has the potential to be very successful; however, it is still in its early stage, and further studies will be necessary to fully exploit its potential. The effectiveness of enzyme EOR has been studied a great deal, and there is a general acknowledgment of its positive effects; however, most of these studies are not comprehensive enough to provide a good understanding of the process. Some experiments are discussed in this article to understand the concept of EEOR, but there is still much to learn about the oil recovery process using enzymes.

### 2.2 Pre-Treatment of Chemicals

Many enzymes have the capability of tolerating the oil and gas reservoir environment. Current uses of enzymes include pre-treatment of biopolymers, gel breaking, desulphurization, and acid production (Harris and McKay, 1998). To improve the capability to form solutions of some biopolymers such as starch, xanthan, guar, lignin, and cellulose pretreatment with enzymes is widely used (Marguerite et al., 1983; Carter and Srivastava, 1987). Furthermore, enzymes can be used as gel breakers (Gupta et al., 1993; Satyanarayana Gupta and Prasek, 1995), For instance, adding enzymes with the drilling mud to selectively degrade one or more polymeric organic viscosifiers therein (Shell and Hitzman, 1992). Enzymes can be used to break and shorten the polymer “backbone”, which results in reducing the viscosity of the fluids (Tjon Joe Pin et al., 1993).

Enzymes can be used for the removal of certain chemicals and generate desired chemicals *in situ*. In a study by Almond et al., they developed a method to use a biologically generated acid as a stimulation fluid to remove drilling damage. The biologically generated acid can be injected into the formation to remove new wellbore damage in carbonate formation. The compatibility of the biologically generated acid with a variety of commonly used oil and water drilling muds has been evaluated. They concluded that the enzyme-based organic acid demonstrated that it is effective in dissolving carbonate core material in the presence of both water- and oil-based muds (Almond et al., 1995).

### 2.3 Plugging of Fractures

The injection and long-term storage of carbon dioxide and nuclear waste mainly depend on the suitable properties of seal and cap rock integrity (Fyfe et al., 1984; Bickle, 2009). Fluid leaking may happen through the high permeability natural fracture zone, induced fractures in the reservoir, or fluid diffusion in cap rock, which is a major concern for safe operations of injection and long-term storage (Shukla et al., 2010; William Carey et al., 2010). Fluid leakage from the subsurface carbon dioxide storage and unconventional oil and gas reservoirs is susceptible to environmental risks (Gasda et al., 2004; Kang et al., 2014). Human health and the subsurface environment might be deteriorated by fluid leakage to the surface or to adjacent active aquifers. Fluid leakage is also a major concern to increased emissions of greenhouse gases, especially in the case of carbon dioxide and methane (Kang et al., 2014). The ozone layer could be formed from the leaked methane gas into the atmosphere, which has an adverse impact on the environment (West and Fiore, 2005).

Permeability of fractures could be controlled using grouting technologies; however, microbially induced calcite precipitation (MICP) has advantages over conventional grouting methods because of its low pressure and low viscosity characteristics. Several studies have been conducted to study MICP as a potential candidate for plugging the fractures and permeability reduction in fractured and porous media. MICP is the advanced and cost-effective technology to plug rock fractures. Safety concerns during and after the oil and gas operations and carbon dioxide injection and storage could be overcome by applying this technology (Whiffin et al., 2007; Tobler et al., 2011; Tobler et al., 2012; Phillips et al., 2013).

A conventional approach used to improve the integrity of the wellbore is the injection of fine cement into the (micro-and macro-scale) fractures, with a minimum aperture size of 120 μm (Harris and Johnson, 1992; Lizak et al., 1992). However, squeezing cement might miss coming leaking locations; therefore, it is somehow difficult to achieve a success rate higher than 50% (Bagal et al., 2016). Several other techniques are also used for plugging fractures with small apertures, such as nanocomposites, epoxies, and gel material (Genedy et al., 2014; Todorovic et al., 2016). However, few concerns are limiting the use of these new techniques such as high pumping pressure being required due to the high viscosity of injection fluids and the chance of missing the fractures with a very fine aperture as compared to the MICP technique. A comparison of a few fracture plugging techniques used for wellbore integrity is shown in Table 1.

**TABLE 1:** Different techniques used for plugging fractures.

Technology	Maturity	Smallest fracture penetrated (μm)	Initial viscosity (cP)	References
Micro-cements	Field use	120–150	250	Stormont, (2016)
Gels and epoxies	Research and few applications in the field	5–50	80–500	Jones et al. (2014)
Ultrafine cement grout	Few applications in the field	150	16–40	Product data sheet
Nanoparticles materials	Research and development stage	<1–13	200	Stormont, (2016)
Microbial induced calcium carbonate precipitation	Research and development stage	2–5	1–3	Phillips, (2013)

A case study was reported by Phillips et al. (2018) in which the MICP process was applied for plugging the potential flow paths or fractures. MICP treatment resulted in a considerable reduction in injection pressure from 0.29 m^3^/h to 0.011 m^3^/h at a depth of 310 m. A reduction in the flow rate at the same surface pressure was observed caused by the MICP treatment. MICP demonstrated high efficacy as it can penetrate the fractures with a micro-sized aperture and tortuous flow paths of potential leakage. Philips et al. reported a field case study for the successful application of the MICP process for plugging the fractures near a wellbore environment of sandstone formation. Injection of urea and calcium chloride solution along with microbial suspension has a positive impact in terms of reduction in the flow rate (from 1.9 to 0.47L/min) and well pressure (from >30 to 7%). Furthermore, fracturing pressure was observed to be considerably increased as compared to the pre-treatment fracturing pressure (Phillips et al., 2016).

Larsen et al. (2006) reported the experimental study of EICP on sand packs. A single solution containing urease, urea, and calcium source was poured in the sand pack. Calcium carbonate precipitation caused the permeability reduction by 25% and attributed unconfined compressive strength up to approximately 10 MPa.

Morkved et al. (2014) conducted laboratory investigations on the consolidation of fractured sand samples collected from an oil field in Norway using CaCO_3_ precipitation. Laboratory experiments were performed for fracture plugging for field implication of the same treatment. During the core flooding experiments, a consolidating solution was injected in three cycles that caused substantial reduction in the permeability after each cycle. Permeability of sand samples was reduced by 25, 35, and 45% after the first, second, and third injection cycles, respectively (Morkved et al., 2014). A case study was reported by Cuthbert et al. for permeability reduction to control groundwater contamination using the MICP process. The enzyme was injected into the high-permeable fractured zones of the subsurface aquifer in the first stage, followed by the solution containing calcium chloride and urea. A significant reduction (35%) in permeability was obtained after the MICP treatment in the fractured rocks, leading to the pollution control of the subsurface aquifers (Cuthbert et al., 2013).

Bansal et al. reported the use of enzyme-based CaCO_3_ precipitation for plugging the unintentionally created fractures connecting injection and production wells to optimize the recovery. The enzyme-based CaCO_3_ precipitation was found to be an effective means of consolidating proppant and plugging fractures in the induced propped fractures. Laboratory experiments were demonstrated to consolidate the sand pack, and plug fractures caused considerable permeability reduction because of enzyme-based calcium carbonate precipitation (Bansal et al., 2014).

Larsen et al. (2008) investigated the use of EICP for plugging the natural and induced fractures. Laboratory experiments were conducted at different conditions to optimize the yield and crystal size of calcium carbonate precipitates for better performance in plugging the fractures in chalk reservoirs. High yield (200 g/L) and larger particles and aggregate size (>1 mm) of calcium carbonate were found to perform better in terms of increasing the plugging strength from 1.0 to 2.9 bar. Moreover, for cost-effective operation for large-scale fracture plugging, low-cost enzyme and stabilizer have been investigated. Hence, the EICP process was found to be an effective technology for plugging the natural and induced fractures (Larsen et al., 2008).

### 2.4 Lost Circulation Material

Lost circulation materials (LCMs) are drilling fluid additives to prevent the fluid flow into the formation. The main cause of fluid losses is drilling through zones with natural fractures, cavernous zones, or a high-permeability matrix (Miller et al., 2013; Alsaba et al., 2014; Gaurina-Međimurec and Pasic, 2014; Alhaidari and Alarifi, 2021). Enzymes can be used to precipitate calcium carbonate or dolomite *in situ*. Precipitation of calcium carbonate can be performed using free enzymes to hydrolyze urea in the presence of a calcium source (Hoang et al., 2019a; Arab, 2019; Hoang et al., 2019b; Putra et al., 2020). In a study conducted by Tariq et al. (2022), modified enzyme-induced calcite precipitation (EICP) solutions were studied and tested to investigate their ability to mitigate lost circulation. They found out that using urea, calcium chloride, magnesium chloride, xanthan gum, and urease enzyme resulted in reducing the permeability by 99% through *in situ* calcite precipitation plugging pores and restricting fluid flow through them. Xanthan gas served as a temperature stabilizer at high temperatures up to 70°C. The multistep process of calcium carbonate precipitation using the modified EICP is as follows (Equations 1–7):

$$\text{CO}{\left({\text{NH}}_{2}\right)}_{2}\;+{\text{ H}}_{2}\text{O }\to {\text{NH}}_{2}\text{COOH }+{\text{ NH}}_{3}\;\text{.}$$ (1)

$${\text{NH}}_{2}{\text{COOH }+\text{H}}_{2}\text{O}\to {\text{NH}}_{3}+{\text{H}}_{2}{\text{CO}}_{3}.$$ (2)

$${\text{H}}_{2}{\text{CO}}_{3}↔{\text{HCO}}_{3}^{-}+{\text{ H}}^{+}.$$ (3)

$$2{\text{NH}}_{3}+2{\text{H}}_{2}\text{O}\to 2NH_{4}^{+}+2\text{OH}\text{.}$$ (4)

$${\text{HCO}}_{3}^{-}+{\text{ H}}^{+}+{2\text{OH}}^{-1}\to {\text{CO}}_{3}^{2-}+2{\text{H}}_{2}\text{O}\text{.}$$ (5)

$${\text{Cacl}}_{2}\left(\text{s}\right)\;\to {\text{Ca}}^{+2}+{\text{Cl}}^{2-}.$$ (6)

$${\text{Ca}}^{+2}\left(\text{aq}\right)+{\text{CO}}_{3}^{2-}\left(\text{aq}\right)\to \text{ CaCO}3\left(\text{S}\right).$$ (7)

In a study conducted by Nemati and Voordouw (2003), they studied plugging unconsolidated porous media using an enzymatic formation of calcium carbonate. In a core-flooding experiment, they showed that Berea sandstone permeability decreased by 98% due to the *in situ* enzymatic formation of calcium carbonate. Overall, *in situ* perception of calcium carbonate has proven to be effective in plugging pore space and therefore reducing permeability. This concept can be applied to mitigate lost circulation issues while drilling by plugging the high-permeable zones, preventing the fluid to flow into the formation.

### 2.5 Sand Consolidation

MICP is a biomediated technique which uses living organisms to catalyze urea of hydrolysis. In the presence of calcium ions, carbonate ions, which is a product of hydrolysis, will lead to the precipitation of calcium carbonate. EICP, on the other hand, does not mimic biological processes but instead uses a biodegradable source such as an enzyme as catalysis for urea hydrolysis. Several drawbacks of MICP, such as sensitivity to pH, temperature, oxygen availability, and pore size, make EICP a preferable option not only for geotechnical application but also for sand consolidation in the oil and gas industry (Khodadadi et al., 2017; Li et al., 2018).

## 3 Enzyme-Induced Calcium Carbonate Precipitation

### 3.1 Enzyme-Induced Calcium Carbonate Precipitation Mechanism

The process works by precipitating CaCO_3_ in the inter-particle contact between sand grains. In order to precipitate calcium carbonate in a controlled manner, calcium and carbonate need to be present in the aqueous solution. Calcium is provided as a salt in the mixing solution, and carbonate is provided by a more complex chemical reaction that will be more delayed and controlled (Larsen et al., 2008). Carbonate is generated by urea breakdown as urea is first hydrolyzed by the biocatalyst enzyme (urease) to ammonia and carbonic acid. Carbonic acid has an equilibrium with carbonate ions and hydrogen carbonate; as the critical concentration is reached, the carbonate will react with calcium precipitating CaCO_3_ (Larsen et al., 2008).

EICP aims to catalyze urea to an extent that precipitation of carbonate is maximized. The products of the catalyzed reaction are carbamate and ammonia. Both are unstable in an aqueous environment such that carbonic acid shifts to the form of bicarbonate and hydrogen ions, while ammonia gives rise to ammonium and hydroxide ions. It is the hydroxide ions that increase the pH level and react with bi-carbamate to produce carbonate ions. The sequence of reactions (Equations 8–12) can be summarized as follows (Zimmer, 2000).

$${\text{H}}_{2}{\text{NCONH}}_{2}{\;+\;2\text{H}}_{2}\text{O }\to {\text{ H}}_{2}{\text{CO}}_{3}{\;+\;2\text{NH}}_{3}.$$ (8)

$${\text{H}}_{2}{\text{CO}}_{3}\;\to {\text{ HCO}}_{3}^{-}{\;+\text{ H}}^{+}.$$ (9)

$${\text{NH}}_{3}{\;+\text{ H}}_{2}\text{O }\to {\text{ NH}}_{4}^{+}{\;+\text{ OH}}^{-}.$$ (10)

$${\text{HCO}}_{3}^{-}{+\text{ OH}}^{-}\to {\text{ CO}}_{3}^{2-}{\;+\text{ H}}_{2}\text{O}\text{.}$$ (11)

$${\text{CaCl}}_{2}\to {\text{Ca}}^{2+}{+\;2\text{Cl}}^{-}.$$ (12)

In the presence of a sufficient amount of calcium ions, the system will yield calcium carbonate precipitation (Equation 13).

$${\text{Ca}}^{2+}{+\text{ CO}}_{3}^{2-}\to {\text{CaCO}}_{3}.$$ (13)

When cement grout containing urea and calcium source is injected together with enzyme into the soil, the final product of reaction which is calcium carbonate will bind particle soils and improve the strength of formation. Enhancement of strength depends on many factors, including precipitation sites.

Laboratory experiments concerned with reaction kinetics are limited to measuring the amount of precipitate as a function of time and temperature. A full reaction kinetic description, however, requires determination of activation energy, Arrhenius constant, the impact of enzyme surface area, etc. Inevitably, for *in situ* CaCO_3_ precipitation, dependency on pressure, temperature, salinity, wellbore, and reservoir fluids, the reaction scheme needs to be elucidated to guide deployment in the field. A holistic kinetic model is a prerequisite for targeted precipitation and to control, among other physical properties, the size of the precipitate to limit near-wellbore permeability reduction. In addition, there is a need to entertain the addition of, for example, magnesium, iron, and manganese to allow for the precipitation of dolomite (CaMg(CO_3_)_2_), ankerite (Ca(Fe,Mg,Mn)(CO_3_)_2_), malganocalcite (Ca,Mn)CO_3_, and ferroan dolomite (FeCO_3_) for three reasons. First, compared to calcium carbonate, dolostones may offer improved physical characteristics such as increased hardness that are preferred for sand control. Second, the vast majority of research pertaining to enzymatic carbonate precipitation used deionized water as a solvent. For field applications, the use of seawater may be more economical, though salinity reportedly has a negative impact on precipitation. In addition to calcium, the most abundant ions of seawater are chloride (Cl^−^), sodium (Na^+^), sulfate (SO_2_^4−^), magnesium (Mg^2+^), and potassium (K^+^), offering alternative reaction pathways for precipitations. Third, some applications may require the precipitant to be softer in nature to help plug particular parts of a formation.

### 3.2 Early EICP Experiments

The efficiency of EICP as grout improvement techniques was first validated by authors Kavazanjian and Hamdan (2015), Nemati and Voordouw (Phillips et al., 2016), and Yashura et al. (Miftah et al., 2020). Nemati and Voordoow used enzymatically produced precipitates for the purpose of improving the performance of water injection practices by eliminating “water fingers”. Since the high-permeability zones lead to large amounts of oil being bypassed, selective plugging of water-encroachment zones can improve oil recovery efficiency. In their studies, test/tube experiments were used to test the increment of cement constituent (urea and Ca), urease concentration, and temperature effect on precipitation. Plugging studies were studies using an experimental set-up consisting of a reactant container, peristaltic pump, columns packed with sand and glass beads, and an effluent container (Nemati and Voordouw, 2003).

The system consisted of three columns which were separated and isolated from each other with clamps. Columns were packed with a mixture of sand and glass beads with a ratio of 70 and 30% weight per weight, respectively. The usage of glass beads helped decrease overall permeability. Before the experiment, distilled water was injected into columns using a peristaltic pump, and the flow rate was measured. Then, the reactant container, which consisted of EICP solution, was injected into one of the columns. The solution was isolated with clamps at a designated temperature at a specified time. After the reaction, distilled water was injected again at the same pressure, and the flow rate was measured. Since the pressure difference applied was the same, permeability comparison could be calculated using Darcy’s Law (Equations 14 and 15):

$$Q=\frac{kAdP}{µL}\;.$$ (14)

$$\frac{Q_{f}}{Q_{i}}=\frac{K_{f}}{K_{i}}\;$$ (15)

The third part of the experiment used a core flooding experiment set up using Berea sandstone. The experimental procedure was similar, where pressure drop before and after treatment with cement grout was measured. Also, after the experiment, the core was cut into pieces and bulk composition was analyzed using XRD and SEM (Nemati and Voordouw, 2003).

In addition to permeability tests, the performance of EICP was further validated by Yashura et al. through uniaxial compressive strength (UCS) tests. To prepare samples, dry sand was mixed with urease powder and pluviated, which ensured uniform distribution of urease. 50 kPa pressure was applied to the pluviated samples in the pressure cell before cement grout injection. The samples were cured for 24 h in pressure and exposed to 150 ml of preflush to clean by-products of chloride and ammonium ions. Prior to the UCS test, some parts of the samples were analyzed using XRD and SEM. The aim of XRD was to identify polymorphs of precipitate, while SEM images showed the shape of calcite crystals and their spatial distribution. Test-tube experiments were further developed by Yashura et al. who also monitored the change of pH during hydrolysis processes. Although pH was not directly indicative of precipitation, its change with respect to time was effective to test the enzyme concentration effect. In addition, inductively coupled plasma atomic emission spectroscopy (ICP-AES) advanced the examination of consumed calcium ions after the experiment (Yasuhara et al., 2012).

Kavazanjian et al. further expanded the geomechanical analysis to the different types of sand characterized by their mean grain size and coefficient of uniformity. In their studies, the treatment method was categorized into injected columns and mix and compact methods. 152 mm-by-51 mm acrylic tubes were first filled with cement grout (urea, urease, CaCl_2_, and stabilizer), and different types of soils such as F-60 sand and Ottawa 20–30 were poured into the tube and compacted thoroughly. The specimens were cured for 30 days, and UCS was tested. On the other hand, for injected columns method, larger PVCs (305 mm by 102 mm) which were filled with sand and tapped, were perforated using a hose which had designated nozzles to inject slurry (Kavazanjian and Hamdan, 2015).

### 3.3 EICP Performance Optimization

The main aim of EICP is to achieve maximum precipitation that, in the end, leads to improved geomechanical properties of rock. The performance of this technique can be evaluated by different factors such as precipitation ratio (PR), unconfined compression strength (UCS), permeability impairment, pH, and electrical conductivity of the cementitious solution.

#### 3.3.1 Performance Metrics

Performance metrics is defined by the actual mass of the precipitant divided by theoretical mass. In these studies, calcium carbonate is the precipitant, and theoretical mass will be ascertained from stoichiometric ratios holding the assumption that all available carbonate ions and calcium sources lead to biomineralization. The mathematical expression is shown in Equations 16 and 17.

$$\text{Precipitation Ratio }\left(\text{PR}\right)=\frac{m_{p}}{m_{t}},$$ (16)

$$m_{t}=CxVxM,$$ (17)

where m_p_ is the mass of the precipitated material, m_t_ is the theoretical mass, C is the concentration of solution in (mol/L), V is the volume of solution, and M is the molar mass of the precipitant.

UCS (unconfined compression strength) is derived from an unconfined compression test (UCT) where a treated rock sample is inserted into a load frame and exposed to uniaxial stress without confining pressure. After analysis of the strain/stress relationship, unconfined compression strength (UCS) is the stress that the sample can withstand till permanent failure can be obtained. Also, Young’s modulus, which represents the degree of deformation to applied stress, and Poisson’s ratio can both be obtained.

pH is a measure of aqueous solution acidity or alkalinity. pH values above 7 indicate an alkaline environment where the relative concentration of hydrogen ions is small. Urea hydrolysis is a reaction where an alkaline environment dominates due to presence of ammonia and carbonate ions. The effect of urease on reaction kinetics was analyzed by Yashuara et al. using pH dynamics (Yasuhara et al., 2012). Also, Miftah et al. correlated their precipitation studies with pH, where a sudden drop in pH values was explained by the creation of nucleation points (Miftah et al., 2020). The reaction kinetics of the EICP solution is presented by van Paassen (2009).

Electrical conductivity is a factor affected by ionic strength or by the presence of ions in solution. Almajed et al. defined two factors to choose an optimum concentration. These two factors were initial and critical electric conductivities. As the concentration of ions increases with electrical conductivity, there would be a threshold value at which PR is affected. Since the addition of enzymes alters the threshold value, this could be used as an appropriate metric to achieve more precipitation (Almajed et al., 2018). Song et al. investigated reaction dynamics considering that the concentration of ions will shrink as precipitation proceeds and that would lead to decrease electrical conductivity, which was further validated by their measurements (Song et al., 2018).

#### 3.3.2 Cement of Constituents Effect and Enzyme Concentration

Test tube experiments are usually carried out to optimize the concentration of cementitious reagents by maximizing the precipitation of calcium carbonate. Commonly, the molar ratio of urea/CaCl_2_ and optimum amount of enzyme for a given molar quantity is of interest. The effect of the enzyme is not only the function of quantity but also an activity which is defined as kU/g (mol of urea hydrolyzed per gram of enzyme). One of the extensive studies was performed by Almajed et al. where molar ratios are varied between 1:1 to 1:2 and enzyme concentration from 1 to 5 g/L. Although increasing enzyme concentration stimulates precipitation, cement quantities above and including 1 M give the least PR (Almajed et al., 2018). This was also validated by Yasuara et al. for 1 g/100 ml enzyme, while Chandra Ravi argued that the threshold value was 0.5 M if enzyme activity was 8 kU/L (Yasuhara et al., 2012; Chandra and Ravi, 2021). As the activity of the enzyme to hydrolyze urea dropped, so does the threshold molar quantity. Carmona et al. reported nearly 60% PR with 4kU/L enzyme when 1 M cement was reacted (Carmona et al., 2016). A test/tube experiment (precipitation test) is illustrated by Neupane et al. which consists of urea–CaCl_2_ mixed with water and urease mixed with water and then filtered, and both are mixed and stirred, and CaCO_3_ is precipitated after 24 h (Neupane et al., 2013).

Increasing enzyme concentration improves precipitation for almost all combinations. However, the pure quantity requires careful selection since it accounts for an estimated 57–98% of the total cost of EICP projects (Abdullah, 2017). Cement reagent with 1 M urea and 1 M CaCl_2_ will yield PR below 40% regardless of 2950 U/g enzyme quantity according to Neupane et al. (2013). PR could reach nearly 70% if an enzyme activity of 40,318 U/g was used (Song et al., 2018). Neupane et al. used the same activity enzyme for 0.5 M cement reagents and reached complete precipitation at 3 g/L.

The effect of molar ratio has been investigated in Hamdan’s experiment through pH measurement where higher (1–6 M) and lower concentrations (0.1–0.6 M) of cement reagents were used. It was concluded that a high ratio of urea to CaCl_2_ degraded the hydrolysis rate due to excessive byproduct NH^4+^ when the concentration is high. For lower molar quantities, this will have a positive effect accompanied by incremental pH (Hamdan, 2015).

The precipitation can also be improved by the addition of Na-montmorillonite. Hua Yuan et al. proved it through pH measurement and calcium ion consumption studies. Urease activity, quantified by the amount of urease hydrolyzed per minute, was higher with 0.2% Na-Mt solution due to the adsorption effect of the additive. This, in turn, leads to faster precipitation and average 0.4 lower pH, which is favorable for the activity of urease (Yuan et al., 2022). Other modifications, such as the replacement of CaCl_2_ by MgCl_2,_ were also a favorable option, and their effectiveness was validated experimentally (Putra et al., 2016; Tariq et al., 2022).

#### 3.3.3 Effect of pH and Temperature

The biomineralization of calcium carbonate with the help of the catalysis of urea by urease is highly temperature-dependent. The effect of temperature on precipitation efficiency has been studied by a few authors. Nemati et al. reported that temperature increase from 20 to 50°C stimulated the production of calcium carbonate from 0.038 to 0.34 g/L/h. Thereafter, a complete conversion to the precipitate was reduced from 300 to 100 h (Nemati and Voordouw, 2003). Al Ahmari et al. studied precipitation of calcium carbonate in different temperatures using two types of solution, with and without enzyme. The temperature range was from 25 to 140°C, and the solution concentration was set based on Almajed et al.’s studies. They concluded that up to 100°C, the enzyme was a prerequisite for urea hydrolysis and that was 140°C temperature that all solutions containing 1 M urea and 0.67 CaCl_2_ gave rise to precipitation regardless of urease concentration (AlAhmari et al., 2020). Although high temperature stimulates hydrolysis of urea, the performance of urease is degraded. Feder et al. investigated different enzyme sources and their catalyzing performance based on the consumption of urea. Jack bean meal (JBM) outperforms other sources (soybean, pigeon pee, and cotton seed) at both temperatures of 30 and 60°C. Stabilization of urea consumption rate was taken as the JBM urease deactivation point, and it differed for different temperatures. Temperatures above 70°C were accompanied by incomplete hydrolysis, while temperature increase from 20 to 65°C showed an incremental change of rate (Feder et al., 2021).

Tariq et al. investigated the thermal stability of cement solution (with xanthan gum) in extreme temperatures up to 800°C. From thermogravimetric analysis, it was found that the main mass loss zone was between 30 and 70°C and highlighted the importance of a temperature stabilizer higher than this temperature (Tariq et al., 2022). Neupane et al. mixed urea and urease at different temperatures and exposed both to room temperature for more than 25 h. Although the final yield of ammonium was the same, evaluation of ammonia was more significant at 25°C compared to 5°C mixing (Neupane et al., 2013).

Other than temperature, maintaining favorable pH conditions is necessary due to several reasons. First, calcium carbonate dissolves in an environment lower than pH 7 (Rebata-Landa, 2007). Also, the shift to carbonate ion formation from acidic form requires a highly alkaline environment which can be supported by hydrolysis of ammonia. However, pH should be increased to the extent that it does not degrade the activity of urease. pH 6.9 is the optimum value for urease according to Chouhan et al. (2018).

### 3.4 EICP to Improve Geomechanical Properties

**FIGURE 2:**
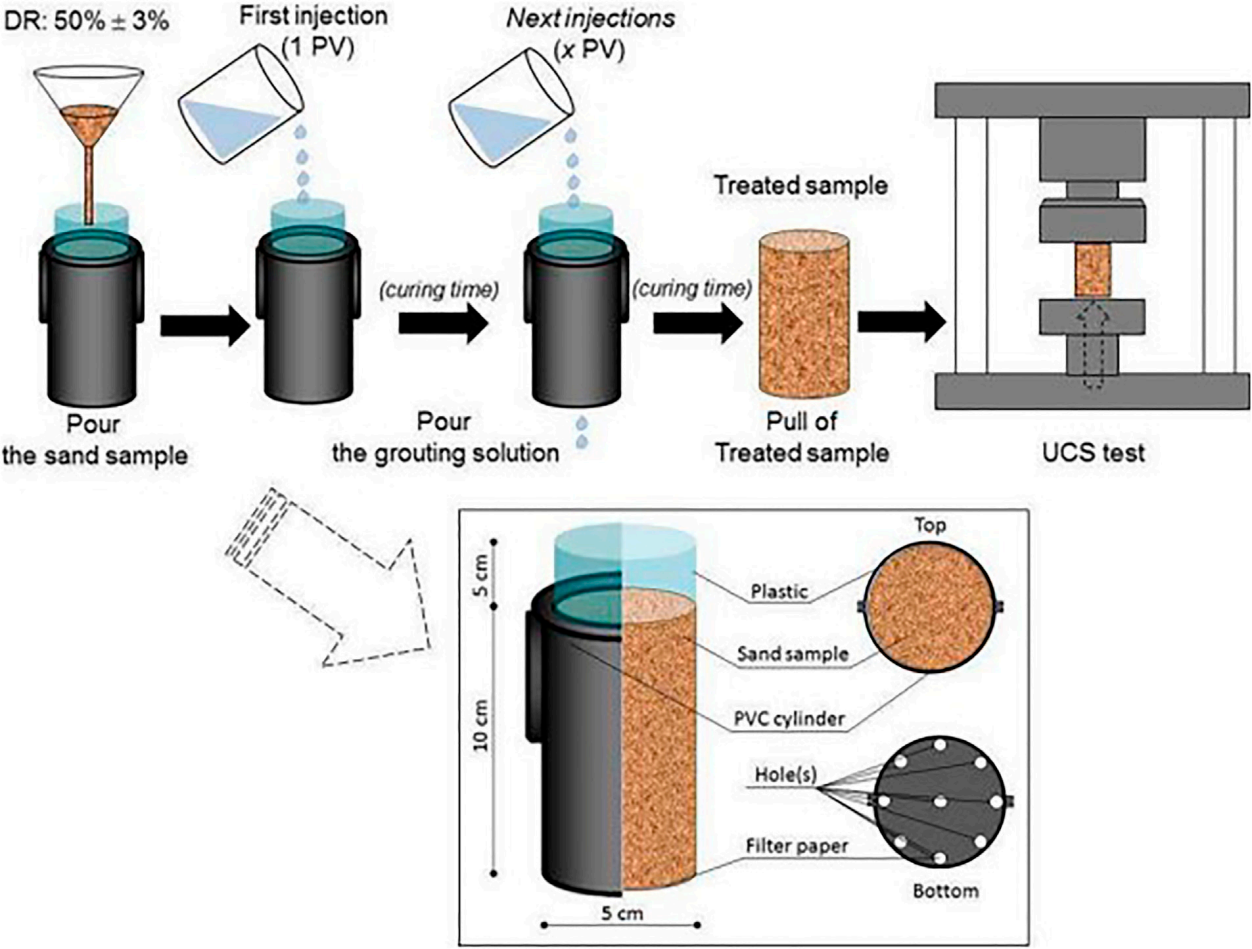
Schematic diagram of treatment and the UCS test (Putra et al., 2017a).

The UCS test is one of the most common methods to measure the strength of samples due to its availability and simplicity. Therefore, many authors use UCS and its correlation with carbonate content as an appropriate metric for their EICP performance evaluation. The role of calcium carbonate is to bind weakly contacted grains in treated samples. However, this precipitation may take place in not only particle contacts but also on the surface of grains. Therefore, the correlation between strength and precipitation amount will be a function of the number of treatment solutions and soil properties in addition to the characteristics of cement grout. The treatment and UCS test schematic diagram are shown in Figure 2 (Putra et al., 2017a).

#### 3.4.1 Carbonate Precipitation and UCS

The primary aim of the EICP consolidation process is to achieve more precipitation since it has a proportional relationship with strength. One single method is to increase the number of treatment cycles which is, in most common cases, measured by the magnitude of pore volume. Before treatment, results are also sensitive to the sample preparation method. If an appropriate method of sand pluviation and relative density is not chosen, the number of treatment cycles will not be effective. Almajed et al. achieved a 10-fold UCS increase from four treatment cycles where Ottawa 20–30 sand was treated with 1 M urea and 0.67 CaCl_2_ (Almajed et al., 2018). The same cement concentration of cement grout and the effect of seawater were studied by Miftah et al. for slightly different sand samples. Their studies observed a nearly 2-fold increase from three treatment cycles reaching to average UCS of 0.263 and 0.224 MPa in the presence of seawater (Miftah et al., 2020). Martin et al. investigated the effect of injected volume given different concentrations. The baseline solution was from Almajed et al. studies. The effect of a 1.5 time more concentrated solution was observed after the second cycle and reached almost 400 kPa difference in the fourth treatment cycle (Martin et al., 2021). Although the magnitude of pore volume as a measure of injected grout is common, quantification through calcite percentage weight or direct weight enhances the analysis of strength. Almajed et al. offered a threshold value of the percentage weight of calcite to drastically increase UCS. However, percentage weight is also prone to errors from non-uniform distribution of calcite during acid digestion measurement, washing out of some precipitates with rinsing water after treatment and human error. Even if there is a direct way to measure calcite precipitation, their correlation will also be affected by the precipitation site.

In another study by Almajed et al., they showed that stabilizers such as non-fat milk yielded comparatively stronger samples for the same amount of calcite. They achieved a value of UCS (higher than 0.5 MPa) that, without stabilizers, requires multiple treatment cycles and 3% calcite (Almajed et al., 2019). Interestingly, selecting an appropriate curing time has a significant effect on UCS. Kee-Lim et al. used clayey sand with different curing times from 7 to 28 days. Solutions, except 3 M urea/CaCl_2_ and 140 ml soybean, showed negligible dependence. UCS increased from 273 to 870 kPa in the curing period for a more concentrated sample. The author correlated it with moisture content where more densely ionized water can slow the dehydration process. In the second part of the experiment, samples were cured at different humidity levels, and it was experimentally proved that UCS depended on humidity, while carbonate content was not affected (Lee and Kim, 2020). The last and most obvious factor affecting UCS and carbonate content relationship is soil type and its heterogeneity which can be characterized by numerous parameters. Studies showed that samples are more responsive to the increment of cement grout and enzyme when it possesses rounded grains and a small number of fine sand. The presence of organic matter and irregular shape had a detrimental effect on treatment (Almajed et al., 2020a).

### 3.5 Effect of Additives on EICP

Original cement grout content is modified by different authors to optimize the performance of EICP. Non-fat milk has been an integral part of EICP solutions which was proved not only to stabilize urease but also used to improve nucleation sites for precipitation. Due to the high cost of pure enzymes, alternatives such as Jack Bean Meal has been offered, which bears similar content to both enzyme and milk solution (Larsen et al., 2008). In addition to UCS improvement, additives are expected to yield stability and viscosity, which improves fluid-retention ability. For this purpose, two different additives, xanthan gum and glycrol, were tested by Pasillas et al. Solution with xanthan gum retained the highest amount of solution and led to uniform distribution of cement through the sample. However, those results were subjective to soil type, where finer-grained samples had all non-uniform distribution with both additives. None of the modified solutions could improve UCS in comparison to the original solution (Pasillas et al., 2018). Hamdan et al. proposed the addition of other biopolymers and hydrogels. The performance of xanthan gum, guar, and polyol cellulose was compared based on penetration depth and vapor pressure measurements. Except for polyol cellulose, all modified solutions decreased the vapor pressure of water to a significant extent. Together with penetration depth studies in soils, these results showed that xanthan gum and guar improved fluid retention abilities in addition to cementation (Hamdan et al., 2016).

Xanthan gum is also used to increase the thermal stability of cement solution, where 1 g/L concentrated solution can stand with only 10% weight loss till 300°C without degrading PR (Tariq et al., 2022). Strength improvement by the additive of sodium alginate (SA) was achieved by Refei et al. According to his conclusions, increment of SA for different moles of cement from 1 to 3 M stimulate consolidation. It was 2 M of cement and 1% SA that make the UCS highest, 1711.4 kPa, caused by the crosslinking ability of biopolymers (Refaei et al., 2020). Na-montmorillonite (Na-Mt) was another potential candidate which is superior to conventional EICP with its content from 2 to 12% in the solution. 8% was selected as the optimum concentration since above that, excess metal ions can degrade urease activity. This also correlated with the swelling ability of Na-Mt which can shrink contact points between grains. Increment of Na-Mt leads to improved surface area and increased contact points, and Na-Mt absorbs water, swells, and degrades the internal structure. It was found that Na-Mt creates paste-like cement within macro-pores. Furthermore, Na-Mt enhanced the UCS and Ca^2+^ utilization ratio (Yuan et al., 2022).

Numerous studies were performed by Putra et al. where precipitation sources were substituted with different minerals. Replacement of CaCl_2_ with either MgSO_4_ or MgCl_2_ improved precipitation weight and thereafter UCS. A more significant effect was observed with MgSO_4_ when 0.04 M or 0.1 of CaCl_2_ was replaced in overall 1 M solution cement. The UCS gap was expanded with treatment cycles and reached 555 kPa (Yuan et al., 2022). Putra et al. extended this study by taking different amounts of MgSO_4_ and analyzing precipitant morphology and mineralogy. In this case, MgSO_4_ varied between 0 and 0.1 M, while there was 1 M of cement and 2 g/L urease. 0.6 Mpa UCS which was 2.5 times higher than conventional EICP achieved with 0.02 or 0.04 M replacement of CaCl_2_. The author explained this trend with the fact that an increased amount of MgSO_4_ stimulates the production of aragonite polymorph (Putra et al., 2017b). The presence of divalent and trivalent ions can also affect the particle size of precipitates, which can enhance the applicability of pore plugging of soils. Although Fe ions cannot yield a significant difference, a favorable ratio of Mg to Ca ions (1:1,000) can give rise to at least two times larger mean crystal size. The same result could be achieved with deficit stoichiometric ratios of either CaCl_2_ or urea. In Jan Larsen et al. study, cement solution concentrated with less CaCl_2_ (20%) increased mean size from less than 100–189 μm, while the opposite effect was observed with excess concentration (Larsen et al., 2008).

Park et al. used calcium sources other than CaCl_2_ which are calcium hydroxide and calcium nitrate. First, it was experimentally validated that increment of all sources increased UCS. However, the rate of improvement for both UCS and precipitation amounts was different, and it was CaCl_2_ which yielded a maximum of 317 kPa at 6.58% weight. It was the solubility of CaCl_2_, which is 100 times higher than that of other sources, that enabled the existence of calcium as ions and in turn more efficient precipitation with carbonate ions (Park et al., 2014).

The core flooding experiment is one of the methods to examine modified contents. Zeeshan et al. prepared different solutions by replacing the source of precipitates and adding xanthan gum. 1 M of urea and a different combination of MgCl_2_ and CaCl_2_ with an overall content of 1 M were tested at room temperature. A higher molar concentration of MgCl_2_ could not yield significant plugging of pores which was correlated with the creation of the least stable precipitate, aragonite. At an elevated temperature of 70 C, the effectiveness of 1 g/L xanthan gum was proved with 99% permeability reduction with 0.9 M CaCl_2_, 0.1 MgCl_2,_ and 3 g/L urease (Tariq et al., 2022). Table 2 summarizes the different additives discussed and their roles.

**TABLE 2:** Effect of different EICP additives.

Sr. No.	Additive name	Effect	References
1	Non-fat milk	Stabilizes urease	Larsen et al. (2008)
		Improves nucleation sites for precipitation	
2	Xanthan gum	Leads to uniform distribution of cement	Pasillas et al. (2018)
		Decreases vapor pressure of water significantly	Hamdan et al. (2016)
		Improves fluid retention abilities	Tariq et al. (2022)
		Increases thermal stability	
3	Glycerol	Increases viscosity between sand particles	Pasillas et al. (2018)
4	Guar	Decreases vapor pressure of water significantly	Hamdan et al. (2016)
		Improves fluid retention abilities	
5	Sodium alginate	UCS improvement	Refaei et al. (2020)
6	Na-montmorillonite	Enhances UCS	Yuan et al. (2022)
		Enhances Ca^2+^ utilization ratio	
7	MgSO_4_	Improves UCS	Putra et al. (2017b)
8	Calcium hydroxide	Improves UCS	Park et al. (2014)
9	Calcium nitrate	Improves UCS	Park et al. (2014)

### 3.6 EICP Performance Assessment

The EICP process has gained attention for its use in geotechnical engineering (Whiffin et al., 2007; DeJong et al., 2010; Burbank et al., 2012). The EICP process requires an appropriate environment in order to achieve the optimum results for its applications (Barabesi et al., 2007; De Muynck et al., 2010). However, the performance of this process is mainly controlled by factors such as soil type, temperatures, pH, curing time, concentration of reactants, and enzyme used. Improper monitoring and controlling of these factors may lead to poor results in the EICP process (Renjith et al., 2020). In addition, the outcome of this biotreatment process also depends on the implementation techniques. Various researchers obtained different results using distinct approaches of implementation.

The effectiveness and efficacy of the EICP process could be assessed using different soil and rock properties such as uniaxial compressive strength (UCS) (Yasuhara et al., 2012; Almajed et al., 2018), triaxial compression strength (Simatupang and Okamura, 2017; Gao et al., 2019), tensile strength (Ahenkorah et al., 2020), and permeability (Yasuhara et al., 2012; Putra et al., 2017a). Furthermore, different analytical approaches, including x-ray diffraction (XRD), scanning electron microscopy (SEM), and energy-dispersive spectroscopy (EDS), are also used to evaluate the morphology and crystal structures of calcium carbonate (CaCO_3_) (Putra et al., 2016). Some of the parameters for the performance assessment of EICP are discussed in the following sections.

#### 3.6.1 Uniaxial Compressive Strength

UCS is the extensively used scale to assess the performance of the EICP process. However, a few discrepancies have been found in the reported literature. Carmona et al. (2016) reported the reduction in the UCS of treated soil with the increase in urea and CaCl_2_ concentrations. On the contrary, Park et al. (2012) reported an increase in UCS of soil by increasing the concentration of urea and CaCl_2_. Oliveira et al. (2017) investigated that the strength of treated soil is not significantly influenced by the change in enzyme concentration (Oliveira et al., 2017). The UCS and CaCO_3_ precipitation are also substantially impacted by the soil packing, EICP treatment cycles, and duration of the EICP treatment (Neupane et al., 2015; Almajed et al., 2018). In addition, UCS is also affected by the factors such as soil grading and particle shape and size (Almajed et al., 2020b).

Various studies reported the UCS after the EICP treatment using different concentrations of urea, CaCl_2_, and enzyme. Table 3 presents the summary of the previously reported UCS along with calcium carbonate content after the EICP treatment of different types of sands.

**TABLE 3:** UCS and calcium carbonate precipitation (%) after EICP treatment reported in the literature.

Sr. No.	Sand Type	Urea (M)	CaCl_2_ (M)	Enzyme (g/L)	Form of enzyme	Avg. CC	Avg. UCS	References
1	River sand	0.10	1.7	1.6	Pure	2.34	0.09	Park et al. (2014)
2	Ottawa F-60	1.38	1.58	0.4	Pure	4.3	0.39	Kavazanjian and Hamdan, (2015)
3	Ottawa 20–30	1.38	1.58	0.4	Pure	2.8	0.43–0.53	Kavazanjian and Hamdan, (2015)
4	River sand	0.10	3.3	1.5	Pure	6.37	0.16	Park et al. (2014)
5	Silica sand	0.5	0.5	1.0	Pure	1.2–8.6	0.04–0.18	Putra et al. (2017b)
6	Toyoura sand	0.5–1.0	0.5–1.0	—	Pure	4.2–7.7	0.37–1.62	Yasuhara et al. (2012)
7	Ottawa 20–30	1.0	0.67	3.0	Pure	1.2–3.6	0.1–1.27	Almajed et al. (2018)
8	River sand	0.10	8.3	1.2	Pure	6.58	0.32	Park et al. (2014)
9	Ottawa 20–30	1.0	0.67	3.0	Pure	1.3	1.7	Khodadadi Tirkolaei et al. (2018)
10	Beach sand	1.0	0.67	—	Pure	1.9–3.7	0.14–0.50	Miftah et al. (2020)
11	Ottawa 20–30	1.0	0.67	3.0	Pure	2.6	0.88	Khodadadi Tirkolaei et al. (2018)
12	Silica sand	1.0	1.0	15	Pure	11–12.4	0.33–0.39	Zhao et al. (2014)
13	Toyoura sand	0.01–0.3	0.75 (Calcium Acetate)	0.005	Crude	—	0.02–0.12	Dilrukshi et al. (2015)
14	Keisha sand	0.50	0.50	0.02	Crude	—	0.06–0.62	Pratama et al. (2021)
15	Silica sand	1.0–3.0	0.67–2.0	3.0	Pure	4.3	1.41	Arab et al. (2021a)
16	Clayey sand	0.75–3.0	0.7–3.0	0.07–0.14	Crude	1.4–5.1	0.27–0.87	Lee and Kim. (2020)
17	Beach sand	0.25–1.0	0.25–1.0	3.0	Pure	5.5	0.77	Muhammed et al. (2021)
18	Ottawa 20–30	1.0	0.67	—	Pure	1.5–1.7	0.29–1.0	Khodadadi Tirkolaei et al. (2020)
19	AI sand	0.50	0.50	0.25	Pure	5.1–17	0.55–4.23	Ahenkorah et al. (2020)
20	Silica sand	1.0	0.67	3.0	Pure	1.6–4.6	0.21–3.0	Rohy et al. (2019)
21	Ottawa 20–30	0.30	0.25	0.85	Pure	0.5–0.7	0.94–1.4	Almajed et al. (2019)
22	Silica sand	1.0	0.67	3.0	Pure	0.8	1.56	Almajed, (2019)
23	Ottawa 20–30	1.0	0.67	3.0	Pure	0.8–1.2	1.65–1.82	Khodadadi Tirkolaei et al. (2018)
24	Silica sand	0.5	0.4	1.0	Pure	2.3–4.2	0.06–0.15	Putra et al. (2017a)

Various studies reported the different values of uniaxial compressive strengths (UCS) and calcium carbonate content (CC) for different types of sands (Table 3). UCS exhibited a positive correlation with CC for all sand types; however, variability in the reported data exists. Data have been classified into two classes: basic EICP (urea, CaCl_2,_ and enzyme) and modified EICP (modified by a biological compound). It has been observed that higher UCS of sand has been reported after the treatment with modified EICP as compared to the basic EICP. Modified EICP treatment has been reported to cause the UCS to increase up to the maximum of 4,230 kPa in some cases, whereas maximum increase in UCS was reported as 1,620 kPa after the treatment with the basic EICP solution, which is almost one-third of what was gained in the case of modified EICP. Basic and modified EICP yields the maximum 8.95 and 17.03% of CC, respectively. Modified EICP can increase the UCS to 2000 kPa even at lower CC (Almajed et al., 2019). One of the reasons for the better performance of modified EICP is the creation of nucleation sites due to the addition of a biological compound and the provision of a favorable environment for the precipitation of CaCO_3_ (Ahenkorah et al., 2021).

In addition, several factors contribute to the change in CC and UCS after the treatment, such as the concentration of reactants (CaCl_2_ and urea), enzyme activity, and the number of treatment cycles (Ahenkorah et al., 2021). There are some variabilities observed in the reported data and trends which might have led to consideration of the size, shape, and density of the sand particles prior to the treatment.

**FIGURE 3:**
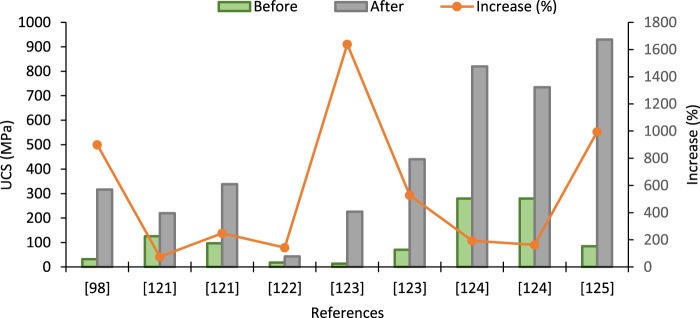
Increase in UCS after EICP treatment sourced from different studies [Data sourced from (Park et al., 2014; Sharma and Ramkrishnan, 2016; Sotoudehfar et al., 2016; Moghal et al., 2020a; Wani and Mir, 2020; Xiao et al., 2020)].

Table 3 indicates that the UCS of the treated soil increases with CC. Initially, UCS was found to be increased gradually at the lower CC, whereas the impact of CC on UCS is more prominent at high CC concentrations for particular types of soils. Variability in the experimental data might be caused due to several reasons, as mentioned earlier. However, the highest UCS is clustered for <2% CaCO_3_ content. Intrinsic properties also contributed to increased UCS and bonding of cementing action of CaCO_3_. In most cases, UCS has decreased at concentrations higher than 5%. A comparison of the reported increase in UCS for different types of soils and under different conditions is shown in Figure 3.

#### 3.6.2 Permeability Change

Many engineering operations require the change of permeability of soil and rocks, such as the plugging of fractures (Nemati and Voordouw, 2003; Nemati et al., 2005). erosion control (Almajed et al., 2020a; Liu et al., 2021), grouting (Yasuhara et al., 2012; Putra et al., 2017b), dust control (Hamdan and Kavazanjian, 2016), and sandstorm mitigation (Miao et al., 2020). Fracture plugging may reduce the permeability by 98% after the precipitation of CaCO_3_ as a result of EICP treatment (Nemati and Voordouw, 2003). Nemati et al. reported a permeability reduction of 62% as a result of a single treatment (Nemati et al., 2005). A significant reduction in sand permeability was reported by Putra et al. (2017b) as a result of EICP treatment. The permeability of the treated Toyoura sand was ten times less than that of the untreated sand (Yasuhara et al., 2012).

**FIGURE 4:**
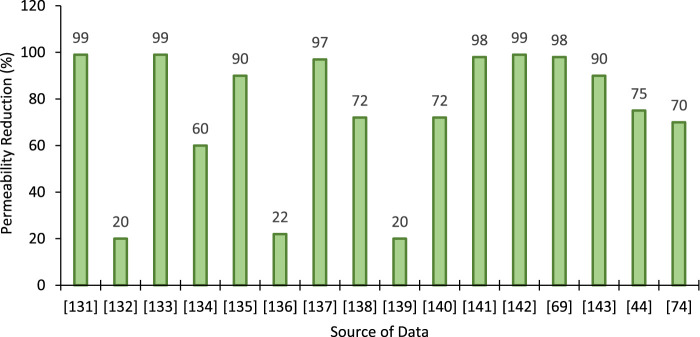
Permeability reduction after EICP treatment reported in the literature [Data sourced from (Cunningham et al., 1991; Ferris and Stehmeier, 1992; Ragusa et al., 1994; Nemati and Voordouw, 2003; Whiffin et al., 2007; Ivanov and Chu, 2008; Van Loosdrecht and Van Paassen, 2009; Yasuhara et al., 2012; Handley-Sidhu et al., 2013; Qabany and Soga, 2014; Ivanov et al., 2010; Soon et al., 2014; Proto et al., 2016; Zamani and Montoya, 2016; Gui et al., 2018; Moghal et al., 2020b)].

It is worth emphasizing that permeability was reduced by different amounts for different soils after the EICP and MICP treatments. One of the primary factors is the amount of CC precipitation, leading to blockage of pores and fractures hindering fluid migration. The reported permeability reductions for different soils after EICP treatment are shown in Figure 4. The permeability of soil depends on various factors that have led to the different degrees of variation after EICP treatment. Furthermore, reactants and enzyme concentrations are different, which also have contributed to the permeability reduction phenomenon.

**FIGURE 5:**
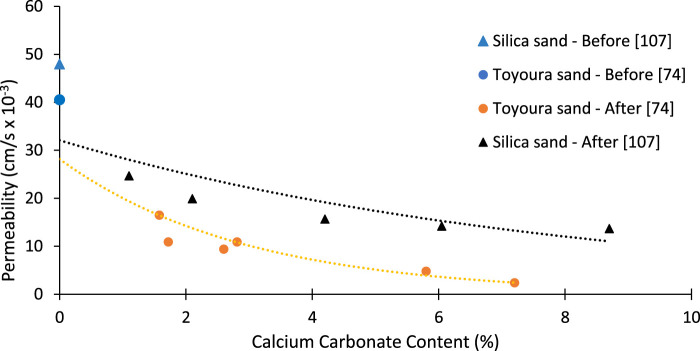
Permeability reduction of Toyoura sand and Silica sand [Data sourced from (Yasuhara et al., 2012; Putra et al., 2017b)].

Silica and Toyoura sands exhibited good harmony with the precipitated calcium carbonate. Permeability decreased significantly with an increase in CC (Figure 5). After the treatment, the sand permeability of Toyoura sand was decreased from 43.7 to 1.53 cm/s for the increase in CC from 0.02 to 7.3%, as shown in Figure 5. For the case of silica sand, a permeability reduction was recorded from 48 to 19.5 cm/s. Permeability reduction trends are different depending upon the soil parameters such as size, sorting, and shape. Pore spaces are filled with precipitated CaCO_3,_ preventing fluid movement (Ahenkorah et al., 2021).

#### 3.6.3 Triaxial Compression Tests

Very few studies have reported the triaxial compression behavior of soils after EICP treatment. The residual and peak strength of soil was considerably increased as a result of the EICP treatment due to precipitation of CaCO_3_. Deviatoric stresses exhibited an increase for the soils with the increase in CaCO_3_ precipitation and vice versa (Ahenkorah et al., 2021). It might be due to the precipitation of CaCO_3_ in the pore spaces and the strengthening of the frame modulus. Furthermore, precipitation caused a noticeable decrease in pore pressure. However, soil with a high degree of precipitation was observed to have the highest pore water pressure under deviatoric stresses because a very small margin was available to the change in void ratio. The changes in deviatoric stress and pore water pressure with CC explain the reduction of peak and residual strength of soils (Ahenkorah et al., 2021).

#### 3.6.4 Mineralogical Composition

X-ray diffraction (XRD) is used to examine and characterize the position of atoms and their arrangement and spacing between the atomic planes (Pingping et al., 2005). Studying the crystal structure and mineral composition of the mineral formation resulting from EICP is a crucial aspect of the performance assessment of such a process. Song et al. used XRD to investigate the mineral composition resulting from MICP on silica sand and found that the resulting mineral contained calcite, vaterite, and quartz. The quartz detected is the mineral component of silica sand. Their XRD analysis indicated that 90% of the CaCO_3-_induced microbial hydrolysis of urea is vaterite (Song et al., 2020).

Tariq et al. examined the created precipitated minerals using modified EICP collected from the test tube experiment using XRD. They found that the resulted precipitation contained 32.7% calcite, 30.1% aragonite, and 35% dolomite (Tariq et al., 2022).

Putra et al. studied the microstructures of the precipitated carbonates using XRD and SEM for performance assessment purposes. They found that increasing the magnesium ratio caused a decrease in the crystal size. Increasing the ratio of magnesium as a substitute material in EICP from 10 to 20% reduced the crystal size by 14% from the original size, and using 50% magnesium reduced the original crystal size by half (Putra et al., 2016).

There are several crystal forms of calcium carbonate, such as calcite, vaterite, and aragonite (Liu, 2020). In a study by He et al., they conducted microscopic analysis using the SEM and identified the crystal types using XRD. They concluded that the calcium carbonate crystal produced is calcite, which is the most stable form of calcium carbonate more than vaterite or aragonite (He et al., 2022).

#### 3.6.5 Molecular Dynamic Simulation

**FIGURE 6:**
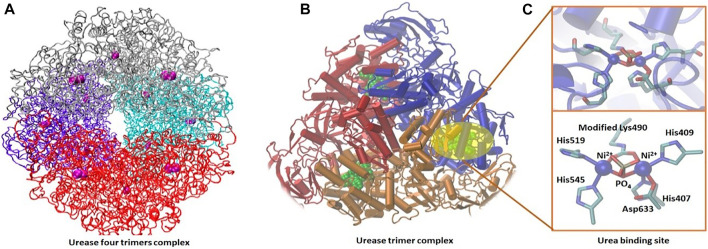
Urease active complex of four trimer subunits **(A)** (Suman et al., 1983) and urease trimer structure **(B)** shown in secondary structure representation with the nickel-active site shown in green. Detail of the nickel-active site is shown in balls and sticks **(C)** (Jabri et al., 1995).

Urease is a ubiquitous enzyme in a large variety of organisms, such as plants, algae, fungi, and prokaryotes. It catalyzes the hydrolytic decomposition of urea into ammonium bicarbonate. Urease is a metalloenzyme, and it uses nickel (Ni^2+^) to catalyze urea hydrolysis (Zambelli et al., 2011). Its structure has two subunits, α and β, and these subunits make up the dimeric αβ subunit. The nickel-binding site is located in the α subunit. Three of these αβ subunits form a trimer of urease that form four trimers, which give the enzyme a total of 24 chains (12 α and 12 β) (Figure 6). The reaction half-life of the urea decomposition by urease is 20 ms, which is 3 × 10^15^ fold than that of all other known hydrolases (Minkara et al., 2014). This indicates the efficiency of urease as a catalyst for urea conversion into ammonium bicarbonate and supports the motivation of using such an efficient enzyme for the precipitation of CaCO_3_, which has a significant impact on the oil recovery from sandstone reservoirs.

The reaction starts with urea hinting *via* the nickel active site. A flexible peptide flap modulates the entrance of the substrate (urea) into the active site cavity (Minkara et al., 2014). This flap has a catalytically essential histidine moving by about 5 Å between the open and closed conformations (Roberts et al., 2012). After a long debate about the urease reaction mechanism, Mazzei et al. recently unraveled the details of the urea conversion mechanism (Mazzei et al., 2019). The authors determined structures of the urease–urea complex in the presence of fluoride which is known to inhibit urease and other intermediates as well. With the analysis of these structures and many other reported x-ray structures, they proposed that the Ni-bridging hydroxide acts as the nucleophile in the reaction, attacking the carbonyl carbon of a urea molecule that chelates the two Ni ions (Mazzei et al., 2019).

Also, the importance of the flexible flap at the border of the active site is evidenced by the directed mutagenesis of C319Y (C: cysteine, Y: tyrosine), which impaired the catalytic activity of the enzyme (Martin and Hausinger, 1992). This cysteine is located in the flexible flap. Such important conformational changes that take place at the level of the urea binding site are a good direction for further investigations that can lead to an explanation of the positive effect induced by the interaction of urease and milk proteins or polymers. It is less likely that the interaction with the aforementioned macromolecules (milk proteins and polymers) provokes a direct catalytic effect on urea hydrolysis. However, the movement of the flexible flap close to the active site could be the reason through which these molecules enhance the enzyme activity at high temperatures. This flap plays an important role in urea binding and the release of the products (ammonium bicarbonate).

β-casein, β-lactoglobulin, and serum albumin proteins are the main proteinaceous components of milk (Pérez-Fuentes et al., 2017). Interestingly, β-casein is well known for its capacity to form micelles in presence of calcium ions (Holt et al., 2013), something that is likely to happen in the presence of different cations existing in an oil reservoir. More intriguingly, β-casein is also known to form a gel at high temperature, which is a positive point in the harsh reservoir conditions. As such, those properties can be used to enhance urease stability and activity. Further studies using molecular dynamic simulation to evaluate and study EICP are highly recommended and could potentially lead to increased understanding of the process at the molecular level for better utilization of the enzymes to precipitate CaCO_3_.

### 3.7 Carbonic Anhydrase Synergistic Role in EICP

Carbonic anhydrases (CAs) are a family of zinc proteins and are known to be involved in the biomineralization of CaCO_3_. In urea hydrolysis, a series of complex reactions are driven by the urease and CA enzymes. Carbonic acid is converted to bicarbonate by CA (Equation 9). CA is well known as a member of the Zn-metalloenzymes that catalyzes the reversible hydration of CO_2_ (Equation 18) (Castro-Alonso et al., 2019; Rodriguez-Navarro et al., 2019).

$${\text{CO}}_{2}{\;+\text{ H}}_{2}\text{O }↔{\text{ HCO}}_{3}^{-}{\;+\text{ H}}^{+}.$$ (18)

Urease and CA seem to play a synergic role in EICP (Botré and Botré, 1989). Studies indicate that the precipitation rate of CaCO_3_ is much higher in bacterial culture than in crude enzyme solutions. In a study by Dhami et al., the authors showed that the calcite precipitation is reduced greatly when both urease and CA are inhibited in comparison with those enzymes individually. CA plays a role in hydrating CO_2_ to bicarbonate, while urease helps in maintaining a pH level for the calcification process (Dhami et al., 2014).

CA plays a major role in the biomineralization of CO_2_ as one of the most economical methods for mitigating global warming (Bose and Satyanarayana, 2017). Thermo-alkali-stable CA, such as Persephonella marina CA (PMCA) and Thermovibrio ammonificans CA (TaCA), were used for biomineralization and resulted in the formation of stable calcite (Jo et al., 2014). Another thermo-alkali-stable CA called Aeribacillus pallidus CA (ApCA) was used to biomineralize, and it caused the precipitation of carbonate ions (Bose and Satyanarayana, 2016).

In a study by Kim et al., they mimicked the natural biomineralization process using CA for the biological conversion of CO_2_ to other chemicals. Recombinant CA has been used to convert CO_2_ into CaCO_3_ economically. They concluded that purified recombinant *Neisseria gonorrhoeae* (NCA) showed a comparable CO_2_ hydration activity to commercial bovine CA (BCA), which greatly promoted formation of solid CaCO_3_ through the acceleration of the CO_2_ hydration rate, which is believed to be slow naturally (Kim et al., 2012).

In a study by Zheng, it was shown that the highest activity of CA was at 30°C, and the highest calcification rate of CaCO_3_ was at a pH level of 9. Therefore, CA can accelerate the hydration of CO_2_ in HCO^3-^ and react with OH^−^ and Ca^2+^ to form CaCO_3_ precipitation in an alkaline environment and in the presence of a calcium source (Zheng, 2019). Moon et al. investigated the effect of different calcium sources with different solubilities and CA enzymes on carbonate mineralization. They concluded that CA improved the overall carbonate mineralization rate, and the reaction rate is highest with CaCl_2,_ followed by Ca(OH)_2_ and CaO (Moon et al., 2020).

## 4 Environmental Impact

The EICP process itself is economical, environment-friendly, and reversible in the sense that if the permeability is impacted drastically by the enzymatic-based solution during the chemical placement process, the acid can be injected and some of the lost permeability can be retained since CaCO_3_ is an acid-soluble material.

Interest in bio-cementation techniques has experienced a sharp increase due to environmental concerns. Prior to the introduction of more eco-friendly techniques, ordinary Portland cement (OPC) was the main source for cementation and ground-improvement technique which aims to improve soil geomechanical properties. OPC, which is a key element in concrete and most construction materials, is considered one of the main contributors to worldwide CO_2_ emissions, and it introduced synthetics and sometimes toxic materials into the environment (Chen et al., 2010; Arab et al., 2021b). Since the production of OPC is a high energy-intensive process, bio-inspired and bio-mediated methods such as MICP and EICP have become major topics of research. Bio-mediated techniques are environmentally friendly and are sustainable to improve the geomechanical properties of soil through microbial-induced calcite precipitation (DeJong et al., 2006; Muhammed et al., 2018).

In oil or gas wells, EICP can be utilized for plugging of fractures or as a lost circulation material. This is carried out to prevent and ensure no uncontrolled fluid or gas leakage occurs during drilling or production. Fluid leakage from the subsurface oil and gas reservoirs is a serious environmental and human health risk. Gas leakage is also a major concern to increased emissions of greenhouse gases, especially in the case of carbon dioxide and methane (Kang et al., 2014). The ozone layer could be formed from the leaked methane gas into the atmosphere, which has an adverse impact on the environment (West and Fiore, 2005).

## 5 Conclusion

The enzyme-induced process can be an economical and viable approach for sand consolidation. EICP can be utilized to solve sand production issues. For this process to be applicable, certain criteria have to be considered, and many researchers have supported that the following criteria should be considered before choosing chemical sand consolidation as a sand control method (Carlson et al., 1992; Wasnik et al., 2005; Chaloupka et al., 2010; Renpu, 2011). The bottom-hole temperature range has to be considered; in general, a range between 100 and 225°F is applicable. The compressive strength has to be taken into consideration as well. Compressive strength values of 600–700 psi can be attained while ensuring 60–90% of the original permeability and yielding about 90% of the original productivity in chemical sand consolidation applications. Another crucial criterion that has to be considered is sand homogeneity. Permeability variations pose problems for successful chemical placement. Chemical penetration in less permeable intervals is difficult to achieve. It has been observed that sand consolidation is less successful in reservoirs with sand production history, though it has been used for remedial jobs and recommended for newly perforated intervals. With all of these criteria being considered, a major concern that has to be assessed is the feasibility of such a process. In general, chemical sand consolidation is economically favorable over conventional gravel packing for short intervals and reservoirs with short production life.

The concept of applying the novel chemical process for controlling sand production may be more effective in terms of strengthening the formation, inhibiting the erosion of sand grains, and, most importantly, increasing the permeability of consolidated sand. This process is expected to yield several advantages compared with other chemical processes. This process may increase the compressive strength of the consolidated sand, while providing high permeability for the movement of reservoir fluids. Hence, production may not be affected. It may hinder the flow of sand particles into the wellbore and enable the unconsolidated or poorly consolidated sand formation to withstand the drag forces generated due to oil production. In addition, mechanical risks can be avoided by applying chemical processes instead of mechanical approaches.

## References

[B1] Abdullah A. (2017). Enzyme Induced Carbonate Precipitation (EICP) for Soil Improvement. Ph.D. dissertation (Arizona State University).

[B2] Ahenkorah I. Rahman M. M. Karim M. R. Beecham S. (2021). Enzyme Induced Calcium Carbonate Precipitation and its Engineering Application: A Systematic Review and Meta-Analysis. Constr. Build. Mater. 308, 125000. 10.1016/j.conbuildmat.2021.125000

[B3] Ahenkorah I. Rahman M. M. Karim M. R. Teasdale P. R. (2020). A Comparison of Mechanical Responses for Microbial- and Enzyme-Induced Cemented Sand. Géotechnique Lett. 10 (4), 559–567. 10.1680/jgele.20.00061

[B4] AlAhmari M. Bataweel M. AlHumam A. AlMajed A. (2020). “Sand Consolidation by Enzyme Mediated Calcium Carbonate Precipitation,” in Paper presented at the Abu Dhabi International Petroleum Exhibition & Conference, Abu Dhabi, UAE, November 2020. 10.2118/203192-MS

[B5] Alhaidari S. A. Alarifi S. A. (2021). Experimental Investigation of Particle Size Degradation and Plugging Efficiency of Three Granular Lost Circulation Materials. Appl. Sci. 11, 9061. 10.3390/app11199061

[B6] Almajed A. Abbas H. Arab M. Alsabhan A. Hamid W. Al-Salloum Y. (2020a). Enzyme-Induced Carbonate Precipitation (EICP)-Based Methods for Ecofriendly Stabilization of Different Types of Natural Sands. J. Clean. Prod. 274, 122627. 10.1016/j.jclepro.2020.122627

[B7] Almajed A. (2019). Enzyme Induced Cementation of Biochar-Intercalated Soil: Fabrication and Characterization. Arab. J. Geosci. 12 (13), 403. 10.1007/s12517-019-4557-z

[B8] Almajed A. Khodadadi Tirkolaei H. Kavazanjian E. (2018). Baseline Investigation on Enzyme-Induced Calcium Carbonate Precipitation. J. Geotech. Geoenviron. Eng. 144 (11), 04018081. 10.1061/(ASCE)GT.1943-5606.0001973

[B9] Almajed A. Lemboye K. Arab M. G. Alnuaim A. (2020b). Mitigating Wind Erosion of Sand Using Biopolymer-Assisted EICP Technique. Soils Found. 60 (2), 356–371. 10.1016/j.sandf.2020.02.011

[B10] Almajed A. Tirkolaei H. K. Kavazanjian E. Hamdan N. (2019). Enzyme Induced Biocementated Sand with High Strength at Low Carbonate Content. Sci. Rep. 9 (1). 10.1038/s41598-018-38361-1 PMC6362242 30718723

[B11] Almond S. W. Harris R. E. Penny G. S. (1995). “SPE 30123. Utilization of Biologically Generated Acid for Drilling Fluid Damage Removal and Uniform Acid Placement across Long Formation Intervals,” in Proceedings of the European Formation Damage Control Conference, The Hague, The Netherlands, 15-16 May 1995, 465–478.

[B12] Alsaba M. Nygaard R. Hareland G. Contreras O. (2014). “Review of Lost Circulation Materials and Treatments with an Updated Classification,” in Proceedings of the AADE Fluids Technical Conference and Exhibition, Houston, TX, USA, 15–16 April 2014.

[B13] Arab M. G. Alsodi R. Almajed A. Yasuhara H. Zeiada W. Shahin M. A. (2021). State-of-the-Art Review of Enzyme-Induced Calcite Precipitation (EICP) for Ground Improvement: Applications and Prospects. Geosciences 11, 492. 10.3390/geosciences11120492

[B14] Arab M. G. Rohy H. Zeiada W. Almajed A. Omar M. (2021). One-Phase EICP Biotreatment of Sand Exposed to Various Environmental Conditions. J. Mat. Civ. Eng. 33 (3), 04020489. 10.1061/(ASCE)MT.1943-5533.0003596

[B15] Arab M. G. (2019). “Soil Stabilization Using Calcium Carbonate Precipitation via Urea Hydrolysis,” in World Congress on Civil, Structural, and Environmental Engineering, Rome, Italy, April 7 - 9, 2019. 10.11159/icgre19.149

[B16] Bagal J. Onadeko G. Hazel P. Dagestad V. (2016). “Annular Barrier as an Alternative to Squeezes in Challenging Wells: Technology Review and Case Histories,” in presented at the SPE/AAPG Africa Energy and Technology Conference, Nairobi City, Kenya, Dec. 2016. 10.2118/AFRC-2583084-MS

[B17] Bansal B. Morkved M. Javadi M. S. Dorthe P. Bähring S. (2014). “Journey of a Technology from Lab to Readiness for Field Application: Enzymatic CaCO3 - A Novel, Environment-Friendly System for Conformance and Sand Consolidation,” in presented at the Abu Dhabi International Petroleum Exhibition and Conference, Abu Dhabi, UAE, November 2014. 10.2118/171958-MS

[B18] Barabesi C. Galizzi A. Mastromei G. Rossi M. Tamburini E. Perito B. (2007). Bacillus Subtilis Gene Cluster Involved in Calcium Carbonate Biomineralization. J. Bacteriol. 189 (1), 228–235. Jan. 2007. 10.1128/JB.01450-06 17085570 PMC1797216

[B19] Bickle M. J. (2009). Geological Carbon Storage. Nat. Geosci. 2 (12), 12. 10.1038/ngeo687

[B20] Bose H. Satyanarayana T. (2017). Microbial Carbonic Anhydrases in Biomimetic Carbon Sequestration for Mitigating Global Warming: Prospects and Perspectives. Front. Microbiol. 8, 1615. 10.3389/fmicb.2017.01615 28890712 PMC5574912

[B21] Bose H. Satyanarayana T. (2016). Suitability of the Alkalistable Carbonic Anhydrase from a Polyextremophilic Bacterium Aeribacillus Pallidus TSHB1 in Biomimetic Carbon Sequestration. Bioprocess. Biosyst. Eng. 39, 1515–1525. 10.1007/s00449-016-1627-4 27215773

[B22] Botré C. Botré F. (1989). Carbonic Anhydrase and Urease: an Investigation *In Vitro* on the Possibility of a Synergic Action. Biochimica Biophysica Acta (BBA)-Protein Struct. Mol. Enzym. 997 (1-2), 111–114. 10.1016/0167-4838(89)90141-6 2502184

[B23] Brooks F. A. (1971). Consolidation of Dirty Sands by Phenol-Formaldehyde Plastic. J. Petroleum Technol. 23, 934–938. 10.2118/3042-PA

[B24] Buckley S. E. Wrightsman G. G. (1945). Two New Chemical Components for Sand Consolidation Techniques. U.S. Patent No. 2,378,817.

[B25] Burbank M. B. Weaver T. J. Williams B. C. Crawford R. L. (2012). Urease Activity of Ureolytic Bacteria Isolated from Six Soils in Which Calcite Was Precipitated by Indigenous Bacteria. Geomicrobiol. J. 29 (4), 389–395. 10.1080/01490451.2011.575913

[B26] Burger J. Bardon C. Gadelle C. (1979). Procede performance de consolidation de formations geologiques. French Patent No. 2 472 658.

[B27] Burger J. G. Gadelle C. P. Marrast J. R. (1986). “Development of a Chemical Process for Sand Control,” in Paper presented at the SPE Annual Technical Conference and Exhibition, New Orleans, Louisiana, October 1986, 1–10. 10.2118/15410-MS

[B28] Butler P. J. (1966). Means of Improving Water Injection Profiles in the Water Flood Program, Rangely Field, ColoradoPaper 875-20-G, API Div. Of Production. Casper, Wyoming.

[B29] Cardwell P. H. Ritch H. B. (1950). Improved Techniques for Application of Plastics in Consolidating Formations. Pamphlet of Dowell, Inc.

[B30] Carlson J. Gurley D. KingPrice-Smith G. C. Water F. (1992). Sand Control: Why and How? Completion/Stimulation Oilfield Rev. 1992, 41–53.

[B31] Carmona J. P. S. F. Oliveira P. J. V. Lemos L. J. L. (2016). Biostabilization of a Sandy Soil Using Enzymatic Calcium Carbonate Precipitation. Procedia Eng. 143, 1301–1308. 10.1016/j.proeng.2016.06.144

[B32] Carter W. H. Srivastava V. K. (1987). Enzymatically-treated Guar Gums. US 4693982.

[B33] Castro-Alonso M. J. Montañez-Hernandez L. E. Sanchez-Muñoz M. A. Macias Franco M. R. Narayanasamy R. Balagurusamy N. (2019). Microbially Induced Calcium Carbonate Precipitation (MICP) and its Potential in Bioconcrete: Microbiological and Molecular Concepts. Front. Mat. 6, 126. 10.3389/fmats.2019.00126

[B34] Chaloupka V. Riyanto L. Tran Q.-B. Rayne A. Kristanto T. Haekal M. (2010). “Remedial Sand Consolidation: Case Study from Mahakam Delta, Indonesia,” in Paper presented at SPE Symposium & Exhibition on Formation Damage, Lafayette, Louisiana, February 2010. (SPE 127489). 10.2118/127489-ms

[B35] Chandra A. Ravi K. (2021). Application of Enzyme-Induced Carbonate Precipitation (EICP) to Improve the Shear Strength of Different Type of Soils. Lect. Notes Civ. Eng. 88, 617–632. 10.1007/978-981-15-6237-2_52

[B36] Chen C. Habert G. Bouzidi Y. Jullien A. (2010). Environmental Impact of Cement Production: Detail of the Different Processes and Cement Plant Variability Evaluation. J. Clean. Prod. 18 (5), 478–485. 10.1016/j.jclepro.2009.12.014

[B37] Chouhan S. Vishnu Priya V. Gayathri R. (2018). Extraction and Partial Purification of Urease Enzyme from Jack Fruit. Int. J. Res. Pharm. Sci. 9 (2), 438–441. 10.26452/ijrps.v9i2.1515

[B38] Cunningham A. B. Characklis W. G. Abedeen F. Crawford D. (1991). Influence of Biofilm Accumulation on Porous Media Hydrodynamics. Environ. Sci. Technol. 25 (7), 1305–1311. 10.1021/es00019a013

[B39] Cuthbert M. O. McMillan L. A. Handley-Sidhu S. Riley M. S. Tobler D. J. Phoenix V. R. R. (2013). A Field and Modeling Study of Fractured Rock Permeability Reduction Using Microbially Induced Calcite Precipitation. Environ. Sci. Technol. 47 (23), 13637–13643. 10.1021/es402601g 24147737

[B40] De Muynck W. De Belie N. Verstraete W. (2010). Microbial Carbonate Precipitation in Construction Materials: A Review. Ecol. Eng. 36 (2), 118–136. 10.1016/j.ecoleng.2009.02.006

[B41] DeJong J. T. Fritzges M. B. Nüsslein K. (2006). Microbially Induced Cementation to Control Sand Response to Undrained Shear. J. Geotech. Geoenvironmental Eng. 132 (11), 1381–1392. 10.1061/(asce)1090-0241(2006)132:11(1381)

[B42] DeJong J. T. Mortensen B. M. Martinez B. C. Nelson D. C. (2010). Bio-mediated Soil Improvement. Ecol. Eng. 36 (2), 197–210. 10.1016/j.ecoleng.2008.12.029

[B43] Dhami N. K. Reddy M. S. Mukherjee A. (2014). Synergistic Role of Bacterial Urease and Carbonic Anhydrase in Carbonate Mineralization. Appl. Biochem. Biotechnol. 172 (5), 2552–2561. 10.1007/s12010-013-0694-0 24407944

[B44] Dilrukshi R. a. N. Watanabe J. Kawasaki S. (2015). Sand Cementation Test Using Plant-Derived Urease and Calcium Phosphate Compound. Mat. Trans. 56 (9), 1565–1572. 10.2320/matertrans.M-M2015818

[B45] Doson J. A. (1922). Oil Well Screen. U.S. Patent No. 1,405,825.

[B46] El-Sayed A. A. Al-Awad M. N. Al-Homadhi E. (2001). Two New Chemical Components for Sand Consolidation TechniquesPresented at Middle East Oil and Gas Show (MEOS). Manama, Bahrain: SPE 6C8225.

[B47] Ereky Karl. (1919). Biotechnologie Der Fleisch-, Fett-, Und Milcherzeugung Im Landwirtschaftlichen Grossbetriebe. Berlin, VII.: Verlag Paul Parey, 84.

[B48] Feder M. J. Akyel A. Morasko V. J. Gerlach R. Phillips A. J. (2021). Temperature‐dependent Inactivation and Catalysis Rates of Plant‐based Ureases for Engineered Biomineralization. Eng. Rep. 3 (2). 10.1002/eng2.12299

[B49] Feng Q.-x. Ni F. Shao D. Ma X.-p. Qin B. Zhou L.-h. (2007). “Eor Pilot Tests with Modified Enzyme in china,” in EUROPEC/EAGE Conference and Exhibition, London, U.K., June 2007 (Society of Petroleum Engineers). 10.2118/107128-MS

[B50] Ferris F. G. Stehmeier L. G. (1992). Bacteriogenic Mineral Plugging. US5143155A. Available: https://patents.google.com/patent/US5143155A/en (Accessed Mar 08, 2022).

[B51] Fyfe W. F. Babuska V. Price N. J. Schmid E. Tsang C. F. Uyeda S. (1984). The Geology of Nuclear Waste Disposal. Nature 310, 537–540. 10.1038/310537a0

[B52] Gao Y. He J. Tang X. Chu J. (2019). Calcium Carbonate Precipitation Catalyzed by Soybean Urease as an Improvement Method for Fine-Grained Soil. Soils Found. 59 (5), 1631–1637. 10.1016/j.sandf.2019.03.014

[B53] Gasda S. E. Bachu S. Celia M. A. (2004). Spatial Characterization of the Location of Potentially Leaky Wells Penetrating a Deep Saline Aquifer in a Mature Sedimentary Basin. Env. Geol. 46 (6), 707–720. 10.1007/s00254-004-1073-5

[B54] Gaurina-Međimurec N. Pašić B. (2014). “Lost Circulation,” in Risk Analysis for Prevention of Hazardous Situations in Petroleum and Natural Gas Engineering. Editors Matanović D. Gaurina-Međimurec N. Simon K.. 1st ed. (Hershey, PA, USA: IGI Global), 73–95.

[B55] Genedy M. Stormont J. Matteo E. Taha M. R. (2014). Examining Epoxy-Based Nanocomposites in Wellbore Seal Repair for Effective CO2 Sequestration. Energy Procedia 63, 5798–5807. 10.1016/j.egypro.2014.11.612

[B56] Gui R. Pan Y. Ding D. Liu Y. Zhang Z. (2018). Experimental Study on Bioclogging in Porous Media during the Radioactive Effluent Percolation. Adv. Civ. Eng. 2018, e9671371. 10.1155/2018/9671371

[B57] Gupta D. V. S. Pakulski M. K. Prasek B. M. (1993). Fracturing Fluid Having a Delayed Enzyme Breaker. US 5226479.

[B58] Hamdan N. (2015). Applications of Enzyme Induced Carbonate Precipitation (EICP) for Soil Improvement. Ph.D. dissertation (Arizona State University).

[B59] Hamdan N. Kavazanjian E. (2016). Enzyme-induced Carbonate Mineral Precipitation for Fugitive Dust Control. Géotechnique 66 (7), 546–555. 10.1680/jgeot.15.P.168

[B60] Hamdan N. Zhao Z. Mujica M. Kavazanjian E. He X. (2016). Hydrogel-Assisted Enzyme-Induced Carbonate Mineral Precipitation. J. Mater. Civ. Eng. 28. 10.1061/(asce)mt.1943-5533.0001604

[B61] Handley-Sidhu S. Sham E. Cuthbert M. O. Nougarol S. Mantle M. Johns M. L. (2013). Kinetics of Urease Mediated Calcite Precipitation and Permeability Reduction of Porous Media Evidenced by Magnetic Resonance Imaging. Int. J. Environ. Sci. Technol. 10 (5), 881–890. 10.1007/s13762-013-0241-0

[B62] Harris K. L. Johnson B. J. (1992). “Successful Remedial Operations Using Ultrafine Cement,” in presented at the SPE Mid-Continent Gas Symposium, Amarillo, Texas, Apr. 1992. 10.2118/24294-MS

[B63] Harris R. E. McKay I. D. (1998). “New Applications for Enzymes in Oil and Gas Production,” in Paper presented at the European Petroleum Conference, The Hague, Netherlands, October 1998. 10.2118/50621-MSUS5566759

[B64] He J. Mao X. Zhou Y. Tang Q. (2022). Cementation of Sand with Enzyme-Induced Carbonate Precipitation (EICP) Using Concrete-Extracted Calcium. Front. Phys. 9, 825356. 10.3389/fphy.2021.825356

[B65] Hoang T. Alleman J. Cetin B. Ikuma K. Choi S.-G. (2019). Sand and Silty-Sand Soil Stabilization Using Bacterial Enzyme-Induced Calcite Precipitation (BEICP). Can. Geotech. J. 56, 808–822. 10.1139/cgj-2018-0191

[B66] Hoang T. Alleman J. Cetin B. Ikuma K. Choi S.-G. (2019). Sand and Silty-Sand Soil Stabilization Using Bacterial Enzyme-Induced Calcite Precipitation (BEICP). Can. Geotech. J. 56, 808–822. 10.1139/cgj-2018-0191

[B67] Holt C. Carver J. A. Ecroyd H. Thorn D. C. (2013). Invited Review: Caseins and the Casein Micelle: Their Biological Functions, Structures, and Behavior in Foods. J. Dairy Sci. 96, 6127–6146. 10.3168/jds.2013-6831 23958008

[B68] Hower W. F. Ramos J. (1957). Selective Plugging of Injection Wells by *In Situ* Reactions. J. Pet. Tech. 9, 17–20. 10.2118/754-g

[B69] Ivanov V. Chu J. (2008). Applications of Microorganisms to Geotechnical Engineering for Bioclogging and Biocementation of Soil *In Situ* . Rev. Environ. Sci. Biotechnol. 7 (2), 139–153. 10.1007/s11157-007-9126-3

[B70] Ivanov V. Chu J. Stabnikov V. He J. Naeimi M. (2010). Iron-Based Bio-Grout for Soil Improvement and Land Reclamation, 7.

[B71] Jabri E. Carr M. Hausinger R. Karplus P. (1995). The Crystal Structure of Urease from Klebsiella Aerogenes. Science 268, 998–1004. 10.1126/science.7754395 7754395

[B72] Jo B. H. Seo J. H. Cha J. C. (2014). Bacterial Extremo- α-carbonic Anhydrases from Deep-Sea Hydrothermal Vents as Potential Biocatalysts for CO2 Sequestration. J. Mol. Catal. B. Enzym. 109, 31–39. 10.1016/j.molcatb.2014.08.002

[B73] Jones P. J. Karcher J. D. Ruch A. Beamer A. Smit P. Hines S. (2014). “Rigless Operation to Restore Wellbore Integrity Using Synthetic-Based Resin Sealants,” in presented at the SPE/EAGE European Unconventional Resources Conference and Exhibition, Vienna, Austria, Feb. 2014. 10.2118/167759-MS

[B74] Kang M. Kanno C. M. Reid M. C. Zhang X. Mauzerall D. L. Celia M. A. (2014). Direct Measurements of Methane Emissions from Abandoned Oil and Gas Wells in Pennsylvania. Proc. Natl. Acad. Sci. U.S.A. 111 (51), 18173–18177. 10.1073/pnas.1408315111 25489074 PMC4280601

[B75] Kavazanjian E. Hamdan N. (2015). Enzyme Induced Carbonate Precipitation (EICP) Columns for Ground Improvement. Geotech. Spec. Publ. 2015, 2252–2261. 10.1061/9780784479087.209

[B76] Khodadadi Tirkolaei H. Javadi N. Krishnan V. Hamdan N. Kavazanjian E. (2020). Crude Urease Extract for Biocementation. J. Mat. Civ. Eng. 32 (12), 04020374. 10.1061/(ASCE)MT.1943-5533.0003466

[B77] Khodadadi Tirkolaei H. Krishnan V. Martin K. Hamdan N. Kavazanjian E. (2018). “Variation in Strength of EICP Treated “Standard” Sand,” in B2G Atlanta 2018, Atlanta, US.

[B78] Khodadadi T. Kavazanjian E. Van Paassen L. Dejong J. (2017). “Bio-Grout Materials: A Review,” in Presented at the 5th International Conference on Grouting, Deep Mixing, and Diaphragm Walls, Grouting 2017, Honolulu, United States, 9-12 July. 2017. 10.1061/9780784480793.001

[B79] Kim I. G. Jo B. H. Kang D. G. Kim C. S. Choi Y. S. Cha H. J. (2012). Biomineralization-based Conversion of Carbon Dioxide to Calcium Carbonate Using Recombinant Carbonic Anhydrase. Chemosphere 87 (10), 1091–1096. 10.1016/j.chemosphere.2012.02.003 22397838

[B80] King J. A. Harry R. Y. (1964). New Process Reduces Channeling in Waterflood Injection Wells. World oil. 93, 1.

[B81] Larsen J. Poulsen M. Lundgaard T. Agerbæk M. (2008). Plugging of Fractures in Chalk Reservoirs by Enzyme-Induced Calcium Carbonate Precipitation. SPE Prod. Oper. 23 (04), 478–483. 10.2118/108589-PA

[B82] Larsen T. Lioliou M. G. Josang L. O. Ostvold T. (2006). “Quasi Natural Consolidation of Poorly Consolidated Oil Field Reservoirs,” in presented at the SPE International Oilfield Scale Symposium, Aberdeen, UK, May 2006. 10.2118/100598-MS

[B83] Lee S. Kim J. (2020). An Experimental Study on Enzymatic-Induced Carbonate Precipitation Using Yellow Soybeans for Soil Stabilization. KSCE J. Civ. Eng. 24 (7), 2026–2037. 10.1007/s12205-020-1659-9

[B84] Li M. Wen K. Li Y. Zhu L. (2018). Impact of Oxygen Availability on Microbially Induced Calcite Precipitation (MICP) Treatment. Geomicrobiol. J. 35 (1), 15–22. 10.1080/01490451.2017.1303553

[B85] Liu K.-W. Jiang N.-J. Qin J.-D. Wang Y.-J. Tang C.-S. Han X.-L. (2021). An Experimental Study of Mitigating Coastal Sand Dune Erosion by Microbial- and Enzymatic-Induced Carbonate Precipitation. Acta Geotech. 16 (2), 467–480. 10.1007/s11440-020-01046-z

[B86] Liu Y. (2020). Experimental Study on Wind-Breaking and Sand-Fixing Using Soybean Urease Induced Carbonate Precipitation. dissertation (Nanjing: Hohai University).

[B87] Lizak K. F. Zeltmann T. A. Crook R. J. (1992). “Permian Basin Operators Seal Casing Leaks with Small-Particle Cement,” in presented at the Permian Basin Oil and Gas Recovery Conference, Midland, Texas, Mar. 1992. 10.2118/23985-MS

[B88] Luque Alanis P. A. AlSofi A. M. Wang J. Han M. (2015). “Toward an Alternative Bio-Based Sp Flooding Technology: I. Biosurfactant Evaluation,” in SPE Asia Pacific Enhanced Oil Recovery Conference. Society of Petroleum Engineers, Kuala Lumpur, Malaysia, August 2015. 10.2118/174621-MS

[B89] Marguerite R. Michel M. Norbert K. (1983). Enzymatic Clarification Process for Improving the Injectivity and Filterability of Xanthan Gums. US 4416990.

[B90] Martin K. K. Khodadadi Tirkolaei H. Kavazanjian E. (2021). Mid-scale Biocemented Soil Columns via Enzyme-Induced Carbonate Precipitation (EICP). Soils Found. 61 (6), 1529–1542. 10.1016/j.sandf.2021.09.001

[B91] Martin P. R. Hausinger R. P. (1992). Site-directed Mutagenesis of the Active Site Cysteine in Klebsiella Aerogenes Urease. J. Biol. Chem. 267, 20024–20027. 10.1016/s0021-9258(19)88659-3 1400317

[B92] Mazzei L. Cianci M. Benini S. Ciurli S. (2019). The Structure of the Elusive Urease–Urea Complex Unveils the Mechanism of a Paradigmatic Nickel-dependent Enzyme. Angew. Chem. Int. Ed. 58, 7415–7419. 10.1002/anie.201903565 30969470

[B93] Miao L. Wu L. Sun X. (2020). Enzyme-catalysed Mineralisation Experiment Study to Solidify Desert Sands. Sci. Rep. 10 (1), 1. 10.1038/s41598-020-67566-6 32606324 PMC7327045

[B94] Miftah A. Khodadadi Tirkolaei H. Bilsel H. (2020). Biocementation of Calcareous Beach Sand Using Enzymatic Calcium Carbonate Precipitation. Crystals 10 (10), 888–915. 10.3390/cryst10100888 Oct, 2020)

[B95] Miller M. Scorsone J. Whitfill D. L. McDonald M. Miller N. (2013). “The Development of a Geopolymer-Based Pill as an Engineered Solution to Lost Circulation,” in Proceedings of the SPE Annual Technical Conference and Exhibition, Society of Petroleum Engineers (SPE), New Orleans, LA, USA, 30 September–2 October 2013. 10.2118/166123-ms

[B96] Minkara M. Ucisik M. Weaver M. Merz K. Jr (2014). Molecular Dynamics Study of *Helicobacter pylori* Urease. J. Chem. Theory Comput. 10, 1852–1862. 10.1021/ct5000023 24839409 PMC4020587

[B97] Mishra S. Ojha K. (2015). Chemical Sand Consolidation: An Overview. J. Petroleum Eng. Technol. 5 (2), 2231–1785.

[B98] Moghal A. A. B. Lateef M. A. Abu Sayeed Mohammed S. Ahmad M. Usman A. R. A. Almajed A. (2020). Heavy Metal Immobilization Studies and Enhancement in Geotechnical Properties of Cohesive Soils by EICP Technique. Appl. Sci. 10, 21. 10.3390/app10217568

[B99] Moghal A. A. B. Lateef M. A. Mohammed S. A. S. Lemboye K. Chittoori B. C. S. Almajed A. (2020). Efficacy of Enzymatically Induced Calcium Carbonate Precipitation in the Retention of Heavy Metal Ions. Sustainability 12 (17), 17. 10.3390/su12177019

[B100] Moon D. H. Eu J. Lee W. Kim Y. E. Park K. T. Ko Y. N. (2020). Comparison of Reactions with Different Calcium Sources for CaCO3 Production Using Carbonic Anhydrase. Greenh. Gas. Sci. Technol. 10, 898–906. 10.1002/ghg.2007

[B101] Morkved M. L. Knight C. Bhagwan B. Garcia Algora A. Zhuang W. Rohde H. C. (2014). “Chemical Consolidation of Sand Propped Fractures in a Chalk Reservoir Offshore Denmark with Enzymatic Calcium Carbonate Scale,” in presented at the International Petroleum Technology Conference, Doha, Qatar, Jan. 2014. 10.2523/IPTC-17456-MS

[B102] Muhammed A. S. Kassim K. A. Ahmad K. Zango M. U. Chong C. S. Makinda J. (2021). Influence of Multiple Treatment Cycles on the Strength and Microstructure of Biocemented Sandy Soil. Int. J. Environ. Sci. Technol. 18 (11), 3427–3440. 10.1007/s13762-020-03073-5

[B103] Muhammed A. S. Kassim K. A. Uba Zango M. (2018). Review on Biological Process of Soil Improvement in the Mitigation of Liquefaction in Sandy Soil. MATEC Web Conf. 250, 01017. 10.1051/matecconf/201825001017

[B104] Nasiri H. Spildo K. Skauge A. (2009). “Use of Enzymes to Improve Water- Flood Performance,” in International Symposium of the Society of Core Analysts, Noordwijk, The Netherlands, 27-30 September, 2009 (Springer), 27–30.

[B105] Nemati M. Greene E. A. Voordouw G. (2005). Permeability Profile Modification Using Bacterially Formed Calcium Carbonate: Comparison with Enzymic Option. Process Biochem. 40 (2), 925–933. 10.1016/j.procbio.2004.02.019

[B106] Nemati M. Voordouw G. (2003). Modification of Porous Media Permeability, Using Calcium Carbonate Produced Enzymatically *In Situ* . Enzyme Microb. Technol. 33 (Issue 5), 635–642. 10.1016/S0141-0229(03)00191-1

[B107] Neupane D. Yasuhara H. Kinoshita N. Ando Y. (2015). Distribution of Mineralized Carbonate and its Quantification Method in Enzyme Mediated Calcite Precipitation Technique. Soils Found. 55 (2), 447–457. 10.1016/j.sandf.2015.02.018

[B108] Neupane D. Yasuhara H. Kinoshita N. Unno T. (2013). Applicability of Enzymatic Calcium Carbonate Precipitation as a Soil-Strengthening Technique. J. Geotech. Geoenviron. Eng. 139 (12), 2201–2211. 10.1061/(asce)gt.1943-5606.0000959

[B109] Oliveira P. J. V. Freitas L. D. Carmona J. P. S. F. (2017). Effect of Soil Type on the Enzymatic Calcium Carbonate Precipitation Process Used for Soil Improvement. J. Mat. Civ. Eng. 29 (4), 04016263. 10.1061/(ASCE)MT.1943-5533.0001804

[B110] Park S.-S. Choi S.-G. Nam I.-H. (2012). Development of Soil Binder Using Plant Extracts. J. Korean Geotechnical Soc. 28, 67–75. 10.7843/kgs.2012.28.3.67

[B111] Park S.-S. Choi S.-G. Nam I.-H. (2014). Effect of Plant-Induced Calcite Precipitation on the Strength of Sand. J. Mat. Civ. Eng. 26 (8), 06014017. 10.1061/(asce)mt.1943-5533.0001029

[B112] Pasillas J. N. Khodadadi H. Martin K. Bandini P. Newtson C. M. Kavazanjian E. (2018). “Viscosity-Enhanced EICP Treatment of Soil,” in IFCEE 2018, 145–154. 10.1061/9780784481592.015

[B113] Patton D. Ablott W. A. (1981). Well Completion and Workovers, Part 19, the System Approach to Sand Control. Pet. Eng. 53 (13), 156–176.

[B114] Pérez-Fuentes L. Drummond C. Faraudo J. Bastos-González D. (2017). Adsorption of Milk Proteins (β-Casein and β-lactoglobulin) and BSA onto Hydrophobic Surfaces. Materials 10, 893. 10.3390/ma10080893 PMC5578259 28767100

[B115] Phillips A. (2013). Biofilm-induced Calcium Carbonate Precipitation: Application in the Subsurface - ProQuest . https://www.proquest.com/openview/e6c14591a6e02e0720eb42b8a4431163/1?pq-origsite=gscholar&cbl=18750 (accessed Mar 08, 2022).

[B116] Phillips A. J. Cunningham A. B. Gerlach R. Hiebert R. Hwang C. Lomans B. P. (2016). Fracture Sealing with Microbially-Induced Calcium Carbonate Precipitation: A Field Study. Environ. Sci. Technol. 50 (7), 4111–4117. 10.1021/acs.est.5b05559 26911511

[B117] Phillips A. J. Lauchnor E. Eldring J. Esposito R. Mitchell A. C. Gerlach R. (2013). Potential CO2 Leakage Reduction through Biofilm-Induced Calcium Carbonate Precipitation. Environ. Sci. Technol. 47 (1), 142–149. 10.1021/es301294q 22913538

[B118] Phillips A. J. Troyer E. Hiebert R. Kirkland C. Gerlach R. Cunningham A. B. (2018). Enhancing Wellbore Cement Integrity with Microbially Induced Calcite Precipitation (MICP): A Field Scale Demonstration. J. Petroleum Sci. Eng. 171, 1141–1148. 10.1016/j.petrol.2018.08.012

[B119] Pingping S. Shiyi Y. Baorong D. Jie S. Kuiyou S. (2005). “Effects of Sweep Efficiency and Displacement Efficiency during Chemical Flooding on a Heterogeneous Reservoir,” in This paper was prepared for presentation at the International Symposium of the Society of Core Analysts, Toronto, Canada, 21-25 August 2005.

[B120] Pratama G. B. S. Yasuhara H. Kinoshita N. Putra H. (2021). Application of Soybean Powder as Urease Enzyme Replacement on EICP Method for Soil Improvement Technique. IOP Conf. Ser. Earth Environ. Sci. 622 (1), 012035. 10.1088/1755-1315/622/1/012035

[B121] Proto C. J. DeJong J. T. Nelson D. C. (2016). Biomediated Permeability Reduction of Saturated Sands. J. Geotech. Geoenvironmental Eng. 142 (12), 04016073. 10.1061/(ASCE)GT.1943-5606.0001558

[B122] Putra H. Yasuhara H. Erizal S. Fauzan M. (2020). Review of Enzyme-Induced Calcite Precipitation as a Ground-Improvement Technique. Infrastructures 5, 66. 10.3390/INFRASTRUCTURES5080066

[B123] Putra H. Yasuhara H. Kinoshita N. Hirata A. (2017a). Application of Magnesium to Improve Uniform Distribution of Precipitated Minerals in 1-m Column Specimens. Geomech. Eng. 12 (5), 803–813. 10.12989/gae.2017.12.5.803

[B124] Putra H. Yasuhara H. Kinoshita N. Hirata A. (2017b). Optimization of Enzyme-Mediated Calcite Precipitation as a Soil-Improvement Technique: The Effect of Aragonite and Gypsum on the Mechanical Properties of Treated Sand. Crystals 7 (2), 59. 10.3390/cryst7020059

[B125] Putra H. Yasuhara H. Kinoshita N. Neupane D. Lu C.-W. (2016). Effect of Magnesium as Substitute Material in Enzyme-Mediated Calcite Precipitation for Soil-Improvement Technique. Front. Bioeng. Biotechnol. 4. 10.3389/fbioe.2016.00037 PMC4854898 27200343

[B126] Qabany A. A. Soga K. (2014). “Effect of Chemical Treatment Used in MICP on Engineering Properties of Cemented Soils,” in Bio- and Chemo-Mechanical Processes in Geotechnical Engineering (ICE Publishing), 107–115. 10.1680/bcmpge.60531.010

[B127] Ragusa S. R. de Zoysa D. S. Rengasamy P. (1994). The Effect of Microorganisms, Salinity and Turbidity on Hydraulic Conductivity of Irrigation Channel Soil. Irrig. Sci. 15 (4), 159–166. 10.1007/BF00193683

[B128] Rebata-Landa V. (2007). Microbial Activity in Sediments: Effects on Soil Behavior. Ph.D. dissertation (Atlanta, GA: Georgia Institute of Technology).

[B129] Refaei M. Arab M. G. Omar M. Omar M. (2020). Sandy Soil Improvement through Biopolymer Assisted EICP. Geo-Congress 2020. 10.1061/9780784482780.060

[B130] Renjith R. Robert D. J. Gunasekara C. Setunge S. O’Donnell B. (2020). Optimization of Enzyme-Based Soil Stabilization. J. Mat. Civ. Eng. 32 (5), 04020091. 10.1061/(ASCE)MT.1943-5533.0003124

[B131] Renpu W. (2011). Advanced Well Completion Engineering. third edition. New York: Gulf Professional Publishing, 105–109.

[B132] Roberts B. P. Miller B. R. III Roitberg A. E. Merz K. M. Jr (2012). Wide-open Flaps Are Key to Urease Activity. J. Am. Chem. Soc. 134, 9934–9937. 10.1021/ja3043239 22670767 PMC3384008

[B133] Rodriguez-Navarro C. Cizer Ö. Kudłacz K. Ibañez-Velasco A. Ruiz-Agudo C. Elert K. (2019). The Multiple Roles of Carbonic Anhydrase in Calcium Carbonate Mineralization. CrystEngComm 21 (48), 7407–7423. 10.1039/c9ce01544b

[B134] Rohy H. Arab M. Zeiada W. Omar M. Almajed A. Tahmaz A. (2019). “One Phase Soil Bio-Cementation with EICP-Soil Mixing,” in presented at the 4th World Congress on Civil, Structural, and Environmental Engineering, Rome, Italy, April, 2019. 10.11159/icgre19.164

[B135] Salahshoor S. Fahes M. Teodoriu C. (2018). A Review on the Effect of Confinement on Phase Behavior in Tight Formations. J. Nat. Gas Sci. Eng. 51, 89–103. 10.1016/j.jngse.2017.12.011

[B136] Salahshoor S. Gomez S. Fahes M. (2019). “Experimental Investigation on the Application of Biological Enzymes for Eor in Shale Formations,” in Unconventional Resources Technology Conference (URTEC), Denver, Colorado, USAJuly 2019 (Springer), 22–24. 10.15530/urtec-2019-1117

[B137] Satyanarayana Gupta D. V. Prasek B. B. (1995). Method for Fracturing Subterranean Formations Using Controlled Release Breakers and Compositions Useful Therein. US 5437331.

[B138] Scott W. W. (1928). Methods of Completing Wells in Gulf Coast Fields. Galveston, Texas: API Fall Meeting.

[B139] Sharma A. Ramkrishnan R. (2016). Study on Effect of Microbial Induced Calcite Precipitates on Strength of Fine Grained Soils. Perspect. Sci. 8, 198–202. 10.1016/j.pisc.2016.03.017

[B140] Shell F. J. Hitzman D. O. (1992). Enzymatic Decomposition of Drilling Mud. US 5165477.

[B141] Shukla R. Ranjith P. Haque A. Choi X. (2010). A Review of Studies on CO2 Sequestration and Caprock Integrity. Fuel 89 (10), 2651–2664. 10.1016/j.fuel.2010.05.012

[B142] Simatupang M. Okamura M. (2017). Liquefaction Resistance of Sand Remediated with Carbonate Precipitation at Different Degrees of Saturation during Curing. Soils Found. 57 (4), 619–631. 10.1016/j.sandf.2017.04.003

[B143] Song C. Chen Y. Wang J. (2020). Plugging High-Permeability Zones of Oil Reservoirs by Microbially Mediated Calcium Carbonate Precipitation. ACS omega 5 (24), 14376–14383. 10.1021/acsomega.0c00902 32596575 PMC7315417

[B144] Song J. Kim Y. Jaewon J. Sim Y-J. Yun T. (2018). “Microstructure of Bio-Mediated Sand by Enzyme Induced Carbonate Precipitation: Relevance to Physio-Mechanical Properties,” in INTERNATIONAL SOCIETY FOR SOIL MECHANICS AND GEOTECHNICAL ENGINEERING.

[B145] Soon N. W. Lee L. M. Khun T. C. Ling H. S. (2014). Factors Affecting Improvement in Engineering Properties of Residual Soil through Microbial-Induced Calcite Precipitation. J. Geotech. Geoenvironmental Eng. 140 (5), 04014006. 10.1061/(ASCE)GT.1943-5606.0001089

[B146] Sotoudehfar A. R. Mirmohammad sadeghi M. Mokhtari E. Shafiei F. (2016). Assessment of the Parameters Influencing Microbial Calcite Precipitation in Injection Experiments Using Taguchi Methodology. Geomicrobiol. J. 33 (2), 163–172. 10.1080/01490451.2015.1025316

[B147] Springham D. Moses V. Cape R. (1991). Biotechnology, the Science and the Business. Chur, Switzerland: Harwood Academic Publishers.

[B148] Stormont J. (2016). Wellbore Seal Repair Using Nanocomposite Materials. , Albuquerque, NM: University of New Mexico. 10.2172/1337552

[B149] Suman Jr. Ellis R. C. Snyder R. E. (1983). Sand Control Handbook. 2nd Edition. Houston, Texas: Gulf Publishing.

[B150] Tariq Z. Mahmoud M. AlahmariBataweel M. M. Bataweel M. Mohsen A. (2022). Lost Circulation Mitigation Using Modified Enzyme Induced Calcite Precipitation Technique. J. Petroleum Sci. Eng. 210, 110043. 10.1016/j.petrol.2021.110043

[B151] Tjon Joe Pin R. M. Beall B. B. (1996). Method of Degrading Cellulose-Containing Fluids during Completions, Workover and Fracturing Operations of Oil and Gas Wells. WO1996021798.

[B152] Tjon Joe Pin R. M. Brannon H. D. Rickards A. R. (1993). Method of Dissolving Organic Filter Cake Obtained from Polysaccharide Based Fluids Used in Production Operations and Completions of Oil and Gas Wells. US 5247995.

[B153] Tobler D. J. Cuthbert M. O. Greswell R. B. Riley M. S. Renshaw J. C. Handley-Sidhu S. (2011). Comparison of Rates of Ureolysis between Sporosarcina Pasteurii and an Indigenous Groundwater Community under Conditions Required to Precipitate Large Volumes of Calcite. Geochimica Cosmochimica Acta 75 (11), 3290–3301. 10.1016/j.gca.2011.03.023

[B154] Tobler D. J. Maclachlan E. Phoenix V. R. (2012). Microbially Mediated Plugging of Porous Media and the Impact of Differing Injection Strategies. Ecol. Eng. 42, 270–278. 10.1016/j.ecoleng.2012.02.027

[B155] Todorovic J. Raphaug M. Lindeberg E. Vrålstad T. Buddensiek M.-L. (2016). Remediation of Leakage through Annular Cement Using a Polymer Resin: A Laboratory Study. Energy Procedia 86, 442–449. 10.1016/j.egypro.2016.01.045

[B156] Van Loosdrecht M. C. M. Van Paassen L. A. (2009). Biogrout, Ground Improvement by Microbial Induced Carbonate Precipitation. Available: http://resolver.tudelft.nl/uuid:5f3384c4-33bd-4f2a-8641-7c665433b57b (Accessed Mar 08, 2022).

[B157] van Paassen L. (2009). Biogrout, Ground Improvement by Microbial Induced Carbonate Precipitation.

[B158] Wang Y. Kantzas A. Li B. Li Z. Wang Q. Zhao M. (2008). “New Agent for Formation-Damage Mitigation in Heavy-Oil Reservoir: Mechanism and Application,” in SPE International Symposium and Exhibition on Formation Damage Control, Lafayette, Louisiana, U.S.A, February 2008 (Society of Petroleum Engineers). 10.2118/112355-ms

[B159] Wani K. M. N. S. Mir B. A. (2020). Unconfined Compressive Strength Testing of Bio-Cemented Weak Soils: A Comparative Upscale Laboratory Testing. Arab. J. Sci. Eng. 45 (10), 8145–8157. 10.1007/s13369-020-04647-8

[B160] Wasnik A. Mete S. Ghosh B. (2005). “Application of Resin System for Sand Consolidation, Mud-Loss Control and Channel Repairing,” in Paper presented at SPE Symposium on International Thermal Operations and Heavy Oil, Calgary, Alberta. SPE/PS-CIM/CHOA 97771.

[B161] West J. J. Fiore A. M. (2005). Management of Tropospheric Ozone by Reducing Methane Emissions. Environ. Sci. Technol. 39 (13), 4685–4691. 10.1021/es048629f 16053064

[B162] Whiffin V. S. van Paassen L. A. Harkes M. P. (2007). Microbial Carbonate Precipitation as a Soil Improvement Technique. Geomicrobiol. J. 24 (5), 417–423. 10.1080/01490450701436505

[B163] William Carey J. Svec R. Grigg R. Zhang J. Crow W. (2010). Experimental Investigation of Wellbore Integrity and CO_2_-brine Flow along the Casing-Cement Microannulus. Int. J. Greenh. Gas Control 4 (2), 272–282. 10.1016/j.ijggc.2009.09.018

[B164] Wrightsman G. G. (1949). Methods for Consolidation of Sands. U.S. Patent No. 2,476,015.

[B165] Xiao J. Z. Wei Y. Q. Cai H. Wang Z. W. Yang T. Wang Q. H. (2020). Microbial-Induced Carbonate Precipitation for Strengthening Soft Clay. Adv. Mat. Sci. Eng. 2020, e8140724. 10.1155/2020/8140724

[B166] Yasuhara H. Neupane D. Hayashi K. Okamura M. (2012). Experiments and Predictions of Physical Properties of Sand Cemented by Enzymatically-Induced Carbonate Precipitation. Soils Found. 52 (3), 539–549. 10.1016/j.sandf.2012.05.011

[B167] Yuan H. Liu K. Zhang C. Zhao Z. (2022). Mechanical Properties of Na-Montmorillonite-Modified EICP-Treated Silty Sand. Environ. Sci. Pollut. Res. 29, 10332–10344. 10.1007/s11356-021-16442-5 34523088

[B168] Zamani A. Montoya B. M. (2016). “Permeability Reduction Due to Microbial Induced Calcite Precipitation in Sand,” in Geo-Chicago 2016: Sustainability and Resiliency in Geotechnical Engineering, Chicago, Illinois, August 14–18, 2016, 94–103. 10.1061/9780784480120.011

[B169] Zambelli B. Musiani F. Benini S. Ciurli S. (2011). Chemistry of Ni2+ in Urease: Sensing, Trafficking, and Catalysis. Accounts Chem. Res. 44, 520–530. 10.1021/ar200041k 21542631

[B170] Zhao Q. Li L. Li C. Li M. Amini F. Zhang H. (2014). Factors Affecting Improvement of Engineering Properties of MICP-Treated Soil Catalyzed by Bacteria and Urease. J. Mat. Civ. Eng. 26 (12), 04014094. 10.1061/(ASCE)MT.1943-5533.0001013

[B171] Zheng T. (2019). Influencing Factors and Formation Mechanism of CaCO3 Precipitation Induced by Microbial Carbonic Anhydrase. Process Biochem. 91. 10.1016/j.procbio.2019.12.018

[B172] Zimmer M. (2000). Molecular Mechanics Evaluation of the Proposed Mechanisms for the Degradation of Urea by Urease. J. Biomol. Struct. Dyn. 17 (5), 787–797. 10.1080/07391102.2000.10506568 10798524

[B173] Zobell C. E. (1946). Bacteriological Process for Treatment of Oil Bearing Earth Formations. US 2413278.

